# Health literacy of the Deaf community: a scoping review

**DOI:** 10.1093/jdsade/enag017

**Published:** 2026-05-15

**Authors:** Kang Suen Goh, Ryan Chun Chien Yau, Thisari Madhavee Dharmapriya, Uma Devi M Palanisamy, Sabrina Anne Jacob

**Affiliations:** Jeffrey Cheah School of Medicine and Health Sciences, Monash University Malaysia, Sunway, Malaysia; Jeffrey Cheah School of Medicine and Health Sciences, Monash University Malaysia, Sunway, Malaysia; Faculty of Medicine, Heidelberg University, Mannheim, Germany; Jeffrey Cheah School of Medicine and Health Sciences, Monash University Malaysia, Sunway, Malaysia; Jeffrey Cheah School of Medicine and Health Sciences, Monash University Malaysia, Sunway, Malaysia

## Abstract

This scoping review examined health literacy among Deaf populations and identified gaps in current evidence. A systemic search of PubMed, Excerpta Medica Database, Cumulative Index to Nursing and Allied Health Literature, and Cochrane was conducted from database inception to December 31, 2024. English-language quantitative, qualitative, and mixed-methods studies reporting from the perspective of Deaf individuals of any age were included, alongside studies evaluating educational interventions. Data were charted using a standardized extraction framework and analysed thematically. Fifty-seven studies from 14 countries met the inclusion criteria. Four themes emerged: health literacy of Deaf people, factors affecting the health literacy of Deaf people, source of health information, and impact of interventions on health literacy levels. Overall, Deaf people demonstrated poorer health literacy levels than hearing populations, largely due to communication barriers and inaccessible health information. While educational interventions improved knowledge, evidence on their sustained impact remains limited. Findings underscore the need for culturally-competent, sign-language-accessible health education to reduce health inequities.

## Introduction

Health literacy is a person’s ability to access health information and analyze and utilize the information in order to enhance and sustain good health (Nutbeam, 1998). In short, it is the set of skills a person needs to make health decisions and navigate the health care system successfully (Hersh et al., 2015). These skills and needs will differ according to the individual and their experiences (Liu et al., 2020). The health literacy of an individual is not only affected by their own efforts and abilities, but is also a reflection of the accessibility of their local health and social care systems. It includes daily living health competencies such as understanding the prescription dosage and the ability to comprehend appointment dates and information in health pamphlets (American Medical Association, 1999).

Health literacy includes three main aspects: (a) acquiring health and healthcare services-related knowledge; (b) understanding, utilizing, and applying this health information effectively; and (c) maintaining one’s health independently and cooperating with healthcare workers (Liu et al., 2020). Being a health-literate person also means making informed and rational decisions in the best interest of one’s health (McQueen et al., 2007). Generally, a person with a high level of health literacy will have better health outcomes because they tend to make better decisions for their health, attributed to their acquired health knowledge (Baker, 2006). They have also been found to be more adherent to their medication (Kalichman et al., 2008; Osborn et al., 2011; Raehl et al., 2006), had better health behaviors, and were less likely to be involved in health-detrimental behaviors such as smoking and consuming alcohol regularly (Liu et al., 2015; Šulinskaitė et al., 2022). Research has also shown that an increase in health literacy levels is related to an increase in self-efficacy, a positive attitude toward health and a better lifestyle, lower hospitalization rates, better health status, and reduced healthcare expenses (Mancuso, 2008; Manganello, 2008).

Poor health literacy, on the other hand, is related to increased hospitalizations, higher usage of emergency care services, poorer health status, and higher mortality (Berkman et al., 2011). Individuals with poor health literacy were also less likely to know about preventive measures to maintain their health, resulting in them being less likely to be involved in screenings and immunizations, less engaged in physical activity, more likely to have an unhealthy diet, and more likely to be either underweight or obese (Aaby et al., 2017; Bennett et al., 2009; White et al., 2008). Poor health literacy is also associated with poorer mental health outcomes, resulting in higher risks of depression and anxiety (Smith et al., 2013; Tambling et al., 2021), suboptimal self-care practices, a higher tendency to smoke, higher healthcare expenses, and higher mortality rates (Baker et al., 1997; Eckman et al., 2012; Jayasinghe et al., 2016; Moser et al., 2015).

Deaf individuals were found to be nearly seven times more likely than typically hearing individuals to have inadequate health literacy (McKee et al., 2015). Deaf with the uppercase “D” refers to a cultural and linguistic minority community of deaf and hard-of-hearing individuals who use sign language as the primary mode of communication and share a common culture, including knowledge, beliefs, and practices (Padden & Humphries, 1988). Conversely, a lowercase “d” for deafness suggests an individual that has hearing loss after learning to speak orally to interact with the world, and who does not consider themselves part of the Deaf community (Fileccia, 2011; Padden & Humphries, 1988).

Deaf individuals experience many difficulties and barriers when accessing health information, including limited information available in sign language, as well as challenges and struggles in navigating and accessing health information on websites in sign language (Jacob et al., 2022; Kushalnagar et al., 2015; Napier & Kidd, 2013). This is compounded by the fact that Deaf people have poorer literacy due to challenges in reading and writing skills (Jacob et al., 2022; Napier & Kidd, 2013; Pollard Jr. & Barnett, 2009). Sign language interpreters are often unavailable during healthcare consultations; however, their presence alone does not guarantee meaningful access or inclusion, as interpreting services are often treated as “quick-fix” solutions that obscure broader structural barriers (Alexander et al., 2012; De Meulder & Haualand, 2021; Reeves et al., 2002). There is also a lack of knowledge among healthcare workers about Deaf people and Deaf culture (Alamro et al., 2023). Indeed, miscommunication is a major issue as healthcare professionals (HCPs) are unaware of the specific communication needs of Deaf people and assume sign language is a visual-representation of the spoken word, when in fact it has its own grammar and syntax, and is informed by local geographical situations and culture (Jacob et al., 2022; World Federation of the Deaf, 2016). This has led to HCPs resorting to writing things down for Deaf people or expecting them to lip-read (Chong et al., 2019; Chong et al., 2021), often leading to miscommunication (Jacob et al., 2021) as most Deaf individuals can also only understand approximately 30% of the content via lip-reading (Nicholls & Mcgill, 1982).

The American Medical Association and the National Institute of Health recommend that health-related educational materials be written at approximately sixth- and eighth-grade reading levels, respectively, while a lower level (around fourth grade) has been suggested for Deaf individuals (National Institutes of Health, 2007; Neuhauser et al., 2013; Weis, 2003). However, most materials are written at a higher level than recommended, with fewer than 5% and 10% meeting the sixth- and eighth-grade readability standards, respectively (Rooney et al., 2021). Deaf populations experience poorer health outcomes across multiple physical and mental health domains compared to hearing populations (Fellinger et al., 2012; Morisod et al., 2022). A systematic review by Rogers et al. (2024) revealed later-stage cancer diagnoses, poorer diabetes control, a higher prevalence of depression and anxiety, increased risk of suicidal thoughts and attempts, and poorer quality of life. These patterns are likely shaped by cumulative barriers to accessible health information, the lack of cultural competency among HCPs, and partial or incomplete solutions being put in place within the healthcare system to meet the needs of the Deaf community (Rogers et al., 2025). In addition, many HCPs are unaware of the health literacy levels of Deaf people and instead overestimate their knowledge regarding certain diseases. As a result, they may assume that Deaf patients have a higher level understanding of medical conditions, disease prevention, and treatment options, leading to fewer efforts to clarify information or explain alternative interventions (Evangelista et al., 2010; Naseribooriabadi et al., 2017).

Given these factors, it would seem necessary to determine the health literacy levels of the Deaf community. Through this study, we aim to identify any disparities or gaps in their health knowledge and use this to provide insights for creating interventions to resolve these problems and ensure equitable access to health information and healthcare services. A scoping review was undertaken to comprehensively map the existing literature on health literacy among Deaf individuals and identify research gaps. Given the diversity of study designs, populations, and conceptualizations of “health literacy”, a systematic review would have been too restrictive and risked excluding relevant studies.

## Methods

The scoping review was performed according to the framework suggested by Arksey and O’Malley and was reported according to the Preferred Reporting Items for Systematic Reviews and Meta-Analyses Statement for Scoping Reviews (PRISMA-ScR) (Appendix A) (Arksey & O’Malley, 2005; Tricco et al., 2018).

### Database search strategy

Two researchers (KSG and RCCY) developed the search keywords based on the study objectives and literature review of relevant studies. The research team and the university librarian were also consulted for further input. A comprehensive search of the keywords related to “Health literacy” and “Deaf Community” was then conducted in the following databases: PubMed, Cumulative Index to Nursing and Allied Health Literature, Excerpta Medica Database, Cochrane Central Register of Controlled Trials, and Cochrane Database of Systematic Reviews. The detailed search strategy can be found in Appendix B. The search results included were from database inception until December 31, 2024. A manual search of Google Scholar and a review of the reference lists of relevant studies were also undertaken to ensure all pertinent research were included.

The search results were exported into EndNote 21 and Covidence, and duplicates were removed. Two researchers (KSG and RCCY) then independently undertook title and abstract screening and full-text screening to select the studies based on the inclusion and exclusion criteria. The two researchers discussed and resolved any discrepancies; if a consensus could not be reached the lead researcher (SAJ) was consulted.

### Study selection

Studies were included if they met the following inclusion criteria (a) reported on health literacy levels from the perspective of Deaf individuals; (b) included deaf sign language users or explicitly identified Deaf participants; (c) employed quantitative, qualitative, or mixed-methods designs; and (d) involved participants of any age group. While not a criteria for inclusion, where a study also reported on the outcomes of educational interventions on health literacy levels, this data was also captured. Studies were excluded if any of the following exclusion criteria were met: (a) studies reporting on the health literacy of deaf participants and/or those with hearing loss, instead of the Deaf with a capital “D”; (b) where studies involved the deaf and/or Deafblind and Deaf, there was no separation of results between the groups; (c) no sign language use indicated, and only stated that participants are hard of hearing; (d) papers were not in English; (e) articles with no abstract or full text available or where the abstract was not available in English; and (f) findings which were not reported from the perspective of Deaf people. The following studies were also excluded: reviews, systematic reviews, meta-analyses, animal studies, in vitro and in vivo studies, conference proceedings and conference abstracts.

### Charting the data

A data extraction form was developed and pilot tested on two articles independently by two researchers (KSG and RCCY). The two researchers then independently extracted the data from all included studies. The following data were extracted: (a) author, year of publication, location of study, title of paper; (b) study objectives and methodology; (c) demographics of the participants (population size, age group, gender, and education level); (d) types of measurement tools used; and (e) findings including key findings or any quantitative data and any reported implications.

### Data analysis

Extracted data were summarized descriptively and thematically. Quantitative findings were tabulated to map study and population characteristics, and health literacy domains. Qualitative findings were analysed using thematic analysis following the approach outlined by Braun and Clarke (2006). This involved familiarization with the data, initial coding, identification and review of themes, and refinement of themes to capture recurring patterns across studies. An iterative process was used during theme development, with themes refined through discussion among the review team to ensure consistency and transparency.

## Results

### Search results

**Figure 1:**
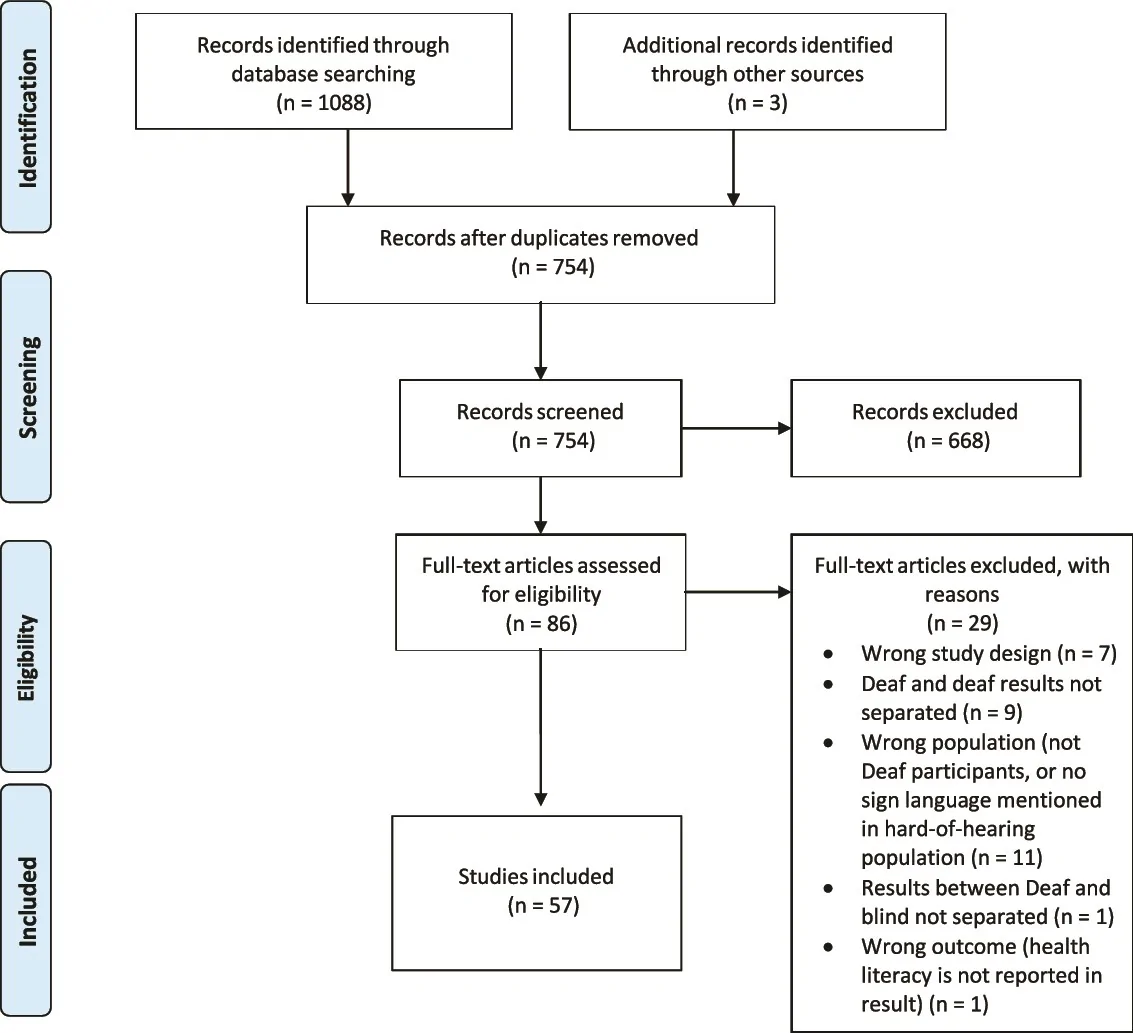
PRISMA-ScR chart showing study selection.

The search identified 1,091 studies. After removing duplicates and upon completion of Title/Abstract screening (*n =* 754), the remaining 86 papers underwent full-text screening, of which 29 studies were excluded, resulting in 57 articles being included in the review. Seven studies were excluded due to the wrong study design, one study was excluded as it included blind participants, nine studies reported findings of signers and non-signers grouped together; therefore, it was not possible to separate the findings and were excluded, and another eleven were excluded as they involved non-signing deaf people (Fig. 1).

### Characteristics of the studies

A total of 6,320 Deaf participants were included across the 57 studies, ranging from 10 to 93 years of age, and with a reported number of 2,484 males (41.6%) and 3,490 females (58.4%). The 57 studies were conducted in 14 different countries, with the most being conducted in the United States (US) (*n =* 31), followed by Ghana (*n =* 4), India (*n =* 3), Turkey (*n =* 3), Saudi Arabia (*n =* 2), Australia (*n =* 2), Indonesia (*n =* 2), Brazil (*n =* 2), Nigeria (*n =* 2), South Africa (*n =* 2), South Korea (*n =* 1, Swaziland (*n =* 1), and the United Kingdom (*n =* 1), while one study was conducted in two different countries i.e., Saudi Arabia and Kuwait. The earliest study included was from 1993 and most studies were published in 2022 (*n =* 7). Seventeen studies explored the effect of interventions on the health literacy levels of Deaf people and the most used study design was cross-sectional (45.6%, *n =* 26), followed by pretest-posttest study design (24.1%, *n =* 13), and qualitative study design (14.8%, *n =* 8).

Different topics/diseases were explored, with 12 each on human immunodeficiency virus (HIV)/acquired immunodeficiency syndrome (AIDS) and cancer, which made up 42.9% of the included papers. Six studies reported on general health literacy, and five on sexual and reproductive health, while four studies each reported on cardiovascular disease (CVD), COVID-19, oral health education (OHE), and women’s health literacy, namely menstrual hygiene, breast examination, and Pap smear. There was one study each on senescence, tobacco, concussion, mental health, dementia, and premarital screening. The summary of included studies are presented in Table 1.

**Table 1:** Summary of the 57 included studies.

Author; publication year; country^a^	Objectives of the study^a^	Population size; Sample characteristics (gender, age)^a^	Study design; Measurement of health literacy; Interventions used^a^	Summary of results^a^
HIV/AIDS literacy (*n* = 12)				
Baker-Duncan et al.; 1997; US	To identify differences in knowledge of AIDS between different grades of students and between males and females.	*n* = 129 Gender = N/A Age = N/A	Cross-sectional design Survey on knowledge of AIDS	Only 9% increase in AIDS knowledge from grade 9 to grade 12 was observed (31% to 40%). Participants were only able to answer three more questions about AIDS correctly by grade 12 versus grade 9 With respect to obtained knowledge, there was no significant difference between male and female participants 60% of survey questions reflected either emerging knowledge or no knowledge, suggesting substantial knowledge is still lacking.
Bat-Chava et al.; 2005; US	To determine the HIV/AIDS knowledge of DHH and barriers to HIV/AIDS education and prevention	*n* = 110; 55 M, 55 F, Age = 15–67 y	Qualitative study with interviews Focus groups and one–one–one interviews using sign language and focus on knowledge level and source of information for HIV/AIDS	Low level of knowledge about transmission, prevention and progression of HIV/AIDS among Deaf adults Deaf adolescents had good basic knowledge about HIV/AIDS and its prevention through their schooling and had more knowledge versus adults Source of HIV/AIDS information for Deaf sign language users: – School, college Friends HOH and oral deaf participants Written materials Deaf adolescents: Television High schools, college, or university Family and friends Preference: Visual media over written information, Less text and more pictures for printed educational materials Educational videos to be presented in sign language Sign language users to provide information in small groups
Bisol et al.; 2008; Brazil	To compare the HIV/AIDS knowledge between Deaf and hearing adolescents	*n* = 92 (42 Deaf, 50 Hearing) Deaf: 21 M, 21 F Hearing: 19 M, 31 F Deaf (x- = 17.8 y (± SD 2.1) Hearing (x- = 16.9 y (± SD 0.8)	Cross-sectional study Computer-assisted self-administered questionnaire on HIV/AIDS knowledge	Hearing students scored significantly higher versus Deaf students (*p* < .001). All hearing students answered ≥8/16 questions correctly; only 47% Deaf answered at least seven questions correctly. In open-ended questions about HIV/AIDS prevention, hearing participants showed higher awareness versus Deaf participants, mentioning condoms more often and providing fewer unclear responses No significant difference in scores between genders
De Andrade et al.; 2010; South Africa	To study knowledge and awareness of HIV/AIDS in Deaf adolescents	*n* = 7 4 M, 3 F; Age = 15–21 y	Qualitative study with interviews One-on-one interviews to explore HIV/AIDS awareness and knowledge	3/7 participants had inaccurate knowledge. Only one participant mentioned having multiple sexual partners as a risk of having HIV 3/7 participants correctly identified unprotected sex as a risk factor Participants were generally aware of the complications of having HIV/AIDS They had some general HIV/AIDS knowledge about the medical and social consequences of HIV/AIDS, life expectancy, but there were gaps in some areas regarding prevention, management and symptoms as well as negative stigma
Doyle.; 1995; US	To explore HIV/AIDS knowledge, attitudes and sexual behavioral norms of the Deaf	*n* = 84; 32 M, 50 F^y^ Age = N/A	Cross-sectional study Questionnaire on general knowledge of HIV	Most participants had relatively high AIDS knowledge scores (mean = 17.3/21, range = 8–21) 52.3% (*n* = 44) scored ≥18 A considerable number of students answered incorrectly on HIV transmission. Through mosquito bites—24% wrong/did not know Donating of blood—41% wrong/did not know Females scored significantly higher versus males (*p* = .079) White and minority groups had no significant difference in knowledge Source of information about sex Friends (most reported at 82%) Printed materials School Television GP/family members (least reported) Source of information about AIDS Printed materials (most reported) Television Friends GPs and family (least reported)
Flowers et al.; 1998; US	To determine AIDS knowledge and attitudes of DHH	*n* = 52 26 M, 26 F x- = 16.65 y, Age = 14–19 y	Cross-sectional study Surveys: AIDS Knowledge and Beliefs Survey	Participants answered correctly to 71.4% of the questions and incorrectly to 12.8% of the statements. Only 63% knew that condom use during sex may protect against AIDS Older age group (age 17–19) scored significantly higher versus younger age group (age 14–16) (*p* < .05) Hard-of-hearing students performed slightly better versus those with no hearing with 72.7% correct answers versus to 64.8%, respectively No significant differences found among participants with different gender and hearing status (presence or absence of hearing)
Goldstein et al.; 2010; US	To evaluate HIV knowledge of Deaf high school students	n = 700 371 M, 329 F; Age = 14–23 y	Cross-sectional study Deaf Adolescent HIV Knowledge and Risk Survey	Students answered correctly to about half (x- = 7.2) of 14 questions (median: 7.0; range = 0–14; SD = 3.8) 70% reported receiving HIV information from school, which had the largest positive effect on HIV knowledge (*p* < .001) Those who identified as Deaf instead of HOH had half a point less of HIV knowledge (*p* < .001) Characteristics that were positively associated with HIV knowledge (*p* < .001) were: Being in a higher school grade Being of White race/ethnicity Reporting having HIV information from school Sources of information School (70%) Internet, newspapers, and magazines (56%) Friends (52%) Family (44%) Television (44%)
Groce et al.; 2007; Nigeria	To determine differences in HIV knowledge between the Deaf and hearing	*n* = 100 (50 DHH, 50 hearing) DHH: 31 M, 19 F Hearing: 31 M, 19 F DHH: ≤ 25 y = 82 26–30 y = 16 31–35 y = 2 36–40 y = 0 ≥ 41 y = 0 Hearing ≤ 25 y = 28 26–30 y = 38 31–35 y = 16 36–40 y = 16 ≥ 41 y = 2	Cross-sectional study Survey on HIV/AIDS knowledge	DHH were significantly more likely (*p* < .05) to believe in inaccurate methods of HIV transmission such as mosquito bites, touching and dirty places DHH were significantly less likely (*p* < .05) to be aware of the potential for mother-to-child HIV transmission (Deaf 52% versus hearing 74%) or transmission through unsterilized needles (Deaf 64% versus hearing 80%) DHH participants tend to report avoiding dirty places and refraining from hugging as a method of HIV prevention Poorer access to information for DHH versus hearing (*p* < .05) DHH: ▪ Posters (17%) ▪ Magazines (22%) ▪ Newspapers (20%) ▪ Community plays (8%) ▪ Co-workers (12%) ▪ Clinics (46%) Hearing: ▪ Posters (72%) ▪ Magazines (76%) ▪ Newspapers (74%) ▪ Community plays (44%) ▪ Co-workers (72%) ▪ Clinics (82%)
Groce et al.; 2006; Swaziland	To establish whether there were measurable differences in the level of knowledge about HIV/AIDS between hearing individuals and individuals who identified themselves as deaf sign language users in Swaziland	*n* = 191 (91 Deaf, 100 hearing) Deaf: 42 M, 49 F Hearing: 43 M, 57 F 18–25 y = 33% 26–30 y = 22% 31–35 y = 22% 36–40 y = 11% 41 + y = 12%	Cross-sectional study Questionnaire on HIV/AIDS	Deaf had significantly poorer knowledge versus hearing (*p* < .05) citing incorrect sources of transmission such as kissing, touching, hugging, mosquito bites, airborne germs, and dirty places Deaf respondents were less familiar with accurate transmission modes like unsterilized needles (Deaf 70%, hearing 89%) and mother-to-child transmission (Deaf 66%, hearing 89%) Deaf participants tend to perceive hand washing and avoiding dirty places as HIV/AIDs preventive methods Deaf respondents incorrectly stated eating healthy foods for prevention (Deaf 73%, hearing 54%) Source of information for Deaf and hearing Deaf ▪ Posters (70%) ▪ Disabled peoples’ organizations (DPOs) (69%) ▪ Television (66%) Hearing ▪ Radio (95%) ▪ Relatives (89%) ▪ Newspapers (79%)
Heuttel et al.; 2001; US	To compare HIV/AIDS knowledge between Deaf and hearing college students	*n* = 80 (34 Deaf, 46 hearing) Deaf: 14 M. 20 F Hearing: 22 M, 24 F Deaf: x- = 25 y, Age = 18–40 y Hearing: x- = 20 y, Age = 18–32 y	Cross-sectional study Questionnaire (HIV/AIDS Knowledge Index)	Deaf students scored significantly lower versus hearing students, 41% of the Deaf students answered at least nine questions correctly versus 91% of hearing students (*p* < .001) 4% hearing students answered seven or fewer questions correctly versus 38% of the Deaf students (*p* < .001) Sources of information Deaf ▪ Friends (88%) ▪ Family (68%) ▪ Reading materials (94%) ▪ Television (94%) ▪ School or teachers (85%) Hearing Friends (51%) Family (44%)
Mprah et al.^u^, 2013; Ghana	To identify HIV/AIDS knowledge among Deaf people	*n* = 178 (152 Survey respondents, 26 focus group) Focus group: 17 M, 9 F, Survey: 87 M, 65 F	Mix method design Focus group interviews and survey on HIV knowledge	Participants in the focus group felt that Deaf individuals had a low level of HIV/AIDS knowledge 98% claimed that the Deaf involve themselves in risky sexual activities due to the lack of awareness about the consequences 62.8% of students and about 34% of adults incorrectly reported that mosquitoes could transmit HIV 56.8% thought HIV/AIDS could be contracted by being around infected individuals There was a statistically significant difference between adults and students in rejecting the misconception that only hearing individuals can contract AIDS (83.9% versus 50%, respectively, *p* = .001). Students were more likely to stigmatize versus adults Many had low HIV prevention knowledge, with 25.7% not knowing that HIV could be transmitted via sharing needles and blades with others
Woodroffe et al.; 1998; US	To compare HIV/AIDS knowledge between DHH and hearing	*n =* 77 (40 DHH, 37 hearing) DHH: 16 M, 24 F Hearing 13 M, 24 F DHH: x- = 43 y Hearing: x- = 46 y	Cross-sectional study Survey on AIDS knowledge	Difference in knowledge observed between the Deaf and hearing DHH individuals more likely to believe that visiting AIDS patients poses a risk of infection (*p* = .01) DHH individuals more likely to believe using public restrooms increases the risk of AIDS (*p* = .01) DHH individuals less likely to identify reducing sexual partners as lowering the risk of AIDS (*p* = .03) DHH individuals less likely to associate sexual contact with drug users as high-risk behavior (*p* = .01) DHH individuals more likely to believe kissing on the cheek increases AIDS risk (*p* = .01) Source of information Magazines (90%) Newspaper (90%) Books (50%) Deaf friends (37.5%). DHH participants tend to receive AIDS information from their friends and printed media versus hearing participants (*p* < .001)
Cancer literacy (*n =* 12)				
Choe et al.; 2009; US	To evaluate the effectiveness of an ASL cervical cancer education video in improving Deaf women’s knowledge level	*n =* 130 x- = 41.23 y (± SD 16.20)	Randomized controlled trial Survey on cancer knowledge Experimental: cervical cancer: catch it early and save your life (cervical cancer education video) Control: National Cancer Institute’s clinical trials PowerPoint education program: “The Basics”	Deaf women who viewed the ASL cervical cancer education video demonstrated significant increases in cervical cancer knowledge immediately after the intervention and retained this knowledge at the 2-month follow-up (*p* < .05) The experimental group scored higher on cervical cancer knowledge questions versus control group Significant gains were noted in five cervical cancer knowledge questions e.g., more participants in the experimental group correctly identified risk factors (97.7% versus 89.5%, *p* = .017) and recognized the importance of the Pap smear (92.9% versus 49.1%, *p* = .000) compared with the control group Only a mild increase in general cancer knowledge post-intervention, which returned to baseline level after 2 months
El Sayed et al.; 2022; Saudi Arabia	To determine whether a health-belief model (HBM) based education intervention helps to improve DHH women’s’ cervical cancer knowledge	*n =* 33; Age = 15–45 y	Pretest-posttest study Quiz on cervical cancer knowledge^b^ Presentations on cervical cancer	Significant improvement in knowledge scores after intervention (*p* = .000) Significant improvements were observed in knowledge about cervical cancer definition, symptoms, risk factors, diagnostic measures, treatment modalities, and preventive measures (*p* < .05 for all) HBM construct scores improved significantly post-intervention (*p* < .05) Number of participants who had poor overall knowledge decreased from 45.6% (pre-intervention) to 12.1% (post-intervention) (*p* = .000) Number of participants who had good overall knowledge improved from 0% (pre-intervention) to 27.3% (post-intervention) Awareness of HPV as the common cause of cervical cancer increased from 30.3% to 81.8% (*p* = .000)
Engelberg et al., 2019; US	To determine the effectiveness of pairing health messages with humor in improving and retaining cancer knowledge in the Deaf community	*n =* 62 30 M, 32 F x- = 31 y (± SD 9.0)	Pretest-posttest study Survey on cancer knowledge Pairing health messages with a joke	Participants’ score improved significantly from pre-intervention (x- = 6.63, SD = 2.16) to post-intervention (x- = 8.84, SD = 2.39), (*p* < .001) Scores after post-intervention and after 1 week of follow-up did not show significant difference, *p* = .312; showing retention of knowledge Awareness of HPV vaccine preventing cervical cancer increased from 41.9% to 80.6% post-test and remained at 75.8% after 1 week (*p* < .05) 58% of participants reported sharing the website and newly acquired knowledge with others and 77% felt motivated to seek additional cancer information
Folkins et al.; 2005; US	To evaluate the effect of an education video on prostate and testicular cancer in improving the health knowledge and access to health information among Deaf men	*n =* 102 102 M x- = 44.35 y (± SD 17.39)	Pretest-posttest study Surveys on testicular and prostate cancer knowledge Educational session using an ASL educational video titled: “Prostate and Testicular Cancer: Know your Options”	Knowledge improved significantly from pre-intervention (x- = 15.2) to immediately after post-intervention (x- = 18.4), *p* < .05. Participants retained the significant improvement in knowledge after the 2-month follow-up (x- = 17.1) versus baseline (x- = 15.3), *p* < .05. Knowledge about screening recommendations for men at average risk (starting at age 50) increased from 65.3% to 93.1% post-video (*p* < .05) Participants’ perception of the Deaf community’s access to health information improved after the video Ranking of best sources of health information (1) Doctors (2) Deaf Community Service (3) Internet (4) Special health education programs Preference: Interactive sources (doctors, Deaf Community Service, friends, and family) over non-interactive sources (pamphlets, internet, and books) Most men (94.1%) believed that there should be more programs on cancer and other health issues for the Deaf community
Harry et al.; 2012; US	To determine the effectiveness of a skin cancer educational video in ASL in improving the Deaf’s knowledge	*n =* 136 (Experiment: 75, Control: 61) 68 M, 68 F 37.56 y (± SD 12.73)	Randomized controlled trial Skin cancer knowledge questionnaire (SCKQ)^c^ Experimental: “Be Smart, Beat Skin Cancer” skin cancer education video in ASL Control: “Cancer Patients and Family Support” video in ASL	The experimental group had a larger increase in SCKQ scores from baseline to post-intervention than the control, *p* < .001. The experimental group had higher mean SCLQ scores post-intervention than the control group, *p* < .001. The experimental group had significant higher knowledge than the control group at 2-month follow-up (*p* < .001). Those who switched from the control to the experimental group had a significant higher knowledge versus pre-intervention score, *p* < .001.
Hickey et al.; 2013; US	To design and evaluate the effectiveness of an educational video in improving breast cancer-related knowledge and screening behaviors among Deaf women	*n =* 122 x- = 45.32 y (± SD 14.19)	Pretest posttest study design Survey on breast cancer Breast cancer education video	25 (21%) reported having no information on breast cancer, while 33 (28%) said they had only a little information Participants had a significantly higher score directly after intervention (8/10 questions) versus pre intervention (3/10 questions) (*p* < .001) Participants had a significantly higher score at 2-month follow-up (6/10 questions) versus pre intervention (3/10 questions) (*p* < .001) A significant association was observed between education level and baseline knowledge, where women with higher education levels scored higher (*p* < .001) More educated women and those with insurance answered more items correctly.
Jensen et al.; 2013; US	To evaluate the effect of video on General, Ovarian, and Total cancer Knowledge scores among the Deaf and to compare the effect between Deaf and hearing women.	*n =* 107 (55 Deaf, 52 Hearing) Deaf: x- = 52 y (± SD 10.4) Hearing: x- = 60 y (± SD 8.1)	Pretest-posttest study design Cancer Knowledge Survey “Finding and Surviving Ovarian Cancer” cancer education video	Deaf women scored significantly lower in baseline general (*p* = .02), ovarian (*p* < .001), and total cancer (*p* < .001) knowledge versus hearing women Both Deaf and hearing participants’ knowledge increased significantly in all domains from baseline to post-intervention (*p* < .001) Hearing participants had a greater change in knowledge from baseline to post-intervention than the Deaf (*p* < .001) The hearing had significantly better post-intervention knowledge than the Deaf (*p* < .001) Deaf women’s post-intervention scores exceeded hearing women’s baseline scores
Kaskowitz et al., 2006; US	To design and evaluate the effect of a prostate cancer education program for Deaf men in ASL	*n =* 121 x- = 40.38 y (± SD 13.91)	Pretest-posttest study design Survey on general and prostate cancer knowledge Educational program in ASL involving a power point presentation	Dissemination of health and cancer prevention/early detection information by HCPs: 14% (17) felt well informed; 37% (45) did not receive any information; 24% (29) received some information but needed other sources to supplement their information in order to feel well informed; 19% (23) received little information or encouragement to ask for more information Participants scored significantly higher post-intervention than at baseline (*p* ≤ .05) Participants scored significantly higher at the 2-month follow-up than at baseline (*p* ≤ .05) Participants who completed college or pursued their education beyond college having significant higher scored at baseline, post-intervention and 2-month follow-up and greater improvement at post-test and 2-month follow-up versus participants with lower education level (*p* < .05) No significant associations between age and knowledge Participants who preferred ASL-based communication demonstrated higher knowledge retention at the 2-month follow-up versus those who preferred English-based methods (e.g., lip-reading, note-writing) Best sources of information: (1) Print sources (58.7%) (2) Physicians (50.4%) (3) Internet (41.3%) Common barriers faced by the Deaf when receiving health information: Communication with HCPs (40%) Inadequate education (31.3%) Inadequate resources such as interpreters (26.1%)
Orsi et al.; 2007; US	To determine cancer screening knowledge, attitudes, and behaviors among the Deaf	*n =* 203 93 M, 100 F Median = 43 y	Cross-sectional study Survey on cancer screening knowledge	Only slightly less than half the participants could define a Pap smear correctly (48%) Participants with an education level higher than high school were more likely to define a Pap smear correctly (*p* < .01) The majority (77%) of women aged above 50 years old could define a mammogram correctly 80% rated the importance of having mammogram a 10/10 69% rated the importance of having colon cancer screening a 10/10 Women who used professional interpreters were more likely to have had a Pap smear in the past 3 years Main source of information Healthcare system (31%) Interpersonal sources (29%) Media (28%)
Sacks et al.; 2013; US	To explore any differences in general and testicular cancer knowledge between Deaf and hearing men before and after watching an education video	*n =* 175 (85 Deaf, 90 hearing); Deaf: x- = 25.75 y (± SD 5.56) Hearing: x- = 22.70 y (± SD 3.46)	Pretest-posttest study design Questionnaire on general and testicular cancer knowledge Prostate and testicular cancer: know your options (cancer educational video in ASL)	Deaf participants scored significantly lower in general, testicular, and total cancer knowledge versus hearing participants (*p* < .01, *p* = .02, *p* < .01, respectively) at baseline The general, testicular, and total cancer knowledge of the Deaf participants increased significantly from baseline to post-intervention (*p* ≤ .001) Deaf participants had a greater change in knowledge versus hearing participants for general cancer knowledge (*p* < .001) but significantly lower change in testicular (*p* < .001) and total cancer (*p* < .001) knowledge than hearing participants Deaf participants scored higher post-intervention versus score of hearing participants at baseline (*p* < .001), suggesting that the gap in knowledge was closed
Sadler et al.; 2001; US	To investigate the impact of a breast cancer education program designed for Deaf women	*n =* 123 x- = 39.3 y (± SD 14.8)	Pretest-posttest study Survey and focus group on breast cancer knowledge Breast cancer education program in ASL.	Except for one respondent, all understood that mammography did not eliminate cancer cells 64.2% (79) correctly recognized that mammography could detect early abnormal change 11.4% reported misinformation that a mammogram could detect abnormalities for the whole upper body and 13% reported a breast biopsy would be performed during a mammogram Participants had an increase in knowledge of monthly BSE, annual clinical breast examinations, and annual mammographies from the age of 40 (*p* < .05) post-intervention Most participants reported they had no knowledge on: Testicular cancer (58.4%) Colorectal cancer (52.0%) Prostate cancer (45.6%) Cervical cancer (36.8%) Ovarian cancer (36.0%) Breast cancer (10.4%) Only 16.2% felt that they receive sufficient health and cancer information from their health providers 18.7% reported they did not receive any information from their doctors Dissemination of health and cancer prevention/early detection information by HCPs: 16.2% (20) felt well informed; 18.7% (23) did not receive any information; 36.6% (45) received some information but needed other sources to supplement their information in order to feel well informed;17% (21) received little information or encouragement to ask for more information Source of health information (1) Printed information (66.7%) (2) Internet (31.7%) (3) Doctors (40.7%) (4) Television (36.6%) (5) Nurses (27.6%) (6) Friends (24.4%) (7) Family (27.6%) (8) School (23.6%)
Yao et al., 2012 ^w^; US	To compare the effectiveness of a cervical cancer educational video between the Deaf and hearing	*n =* 233 (127 Deaf, 106 Hearing) 127 Deaf women, 106 hearing women Deaf: x- = 41.23 y (±SD 16.20), Age = 19–93 y	Pretest-posttest study Survey on cancer knowledge Cervical cancer: catch it early and save your life (cervical cancer education video)	Deaf participants scored significantly lower than the hearing in general, cervical, and total cancer knowledge at the pre-intervention stage (*p* < .001) Deaf participants had a significantly higher change in the mean score in general, cervical and total cancer knowledge at the pre-intervention stage after intervention than the hearing (*p* < .001) The post-intervention knowledge of the Deaf was higher than the baseline knowledge of hearing participants
General health literacy (*n =* 6)				
Choi et al.; 2023; South Korea	To identify linguistic, functional, and internal health literacy among deaf people with hypertension	*n =* 95 34 M, 61 F 64.81 y (± SD 9.83)	Cross-sectional study Questionnaire Linguistic health literacy: Korean Health Literacy Assessment Tool-2 (KHLAT-2)^d^ Functional health literacy: Short Form of the Korean Functional Health Literacy (S-KFHLT)^e^ Internet health literacy: Digital Health Technology Literacy Assessment Questionnaire (DHTL-AQ)^f^	Linguistic health literacy: Most participants scored in the 4–6th grade range (31.6%), followed by the 0–3rd grade range (30.5%) x- ± SD = 33.41 ± 21.25 (out of 66 points) Functional health literacy: x- score = 1.43 ± 2.31 (out of 8 points) Total percentage correct was 17.9% Internet health literacy: Total x- score = 8.72 ± 9.47 (out of 34.00 points)
Gur et al.; 2020; Turkey	To identify health literacy level of DHH adolescents	*n =* 88 55 M, 33 F x- = 16.8 y (± SD 1.10) Age = 15–18 y	Cross-sectional study Turkish Health Literacy Scale (TSY-32)^g^	Inadequate health literacy: 70.5% Limited health literacy: 19.3% Adequate health literacy: 2.3% Excellent health literacy: 0.8% Factors that were positively associated with health literacy (*p* < .05) Age Grade of the students Good family economic status Girls had significantly lower score than boys (*p* < .05) Preference of the Deaf 87.5% prefer sign language when receiving instructions for their medication and treatment 55.7% prefer to have these in writing. 67.0% want publications to be played in sign language 72.7% wished the hospital had onsite translators
Kushalnagar et al.^x^, 2018; US	To explore critical health literacy (CHL) among deaf college students	*n =* 37 17 M, 20 F x- = 22.38 y (± SD 2.68)	Cross-sectional study Functional health literacy: S-TOFHLA in adults^h^ Interactive health literacy: questionnaire^i^ Critical health literacy^j^: Qualitative response after watching a video	Most had adequate functional health literacy (95%) For interactive health literacy, only 30% of the respondents often or always talked about health issues with their family; 41% talked with their friends Frequent health-related discussions with friends were significantly associated with better CHL scores (*p* = *.*025) versus discussions with family which had no significant effect on CHL scores Early exposure to ASL had a strong positive effect on CHL scores (*p* < .05) Criteria for critical health literacy (recommending visiting a doctor, providing support and avoiding misinformation): Met all criteria: 49% Met some criteria: 46% Did not meet the criteria: 5%
McKee et al.; 2015; US	To explore the health literacy level among the Deaf	*n =* 405 (166 Deaf, 209 hearing) Deaf: 72 M, 94 F Hearing 102 M, 137 F Deaf: x- = 54.2 y (± SD 7.9) Hearing x- = 54.0 y (± SD 7.8)	Cross-sectional study Newest vital sign^k^ and Wagner’s heart disease fact questionnaire^l^	Deaf respondents 6.9 times more likely versus hearing participants to have inadequate health literacy (*p* < .001).Deaf participants answered fewer questions correctly versus hearing individuals on heart disease (*p* < .001) Deaf participants’ score on the ASL-NVS were significantly lower versus hearing participants (*p* < .01) Lower education level, income and poorer English reading literacy were significantly associated with inadequate health literacy (*p* < .01) Race or ethnicity (White versus other) (*p* = .401) and gender (male versus female) (*p* = .78) had no significant association with inadequate health literacy
Steinberg et al. 2002; US	To explore the knowledge level, perception and experiences of healthcare among Deaf women	*n =* 45 Age^m^ = 8–67 y, Median = 42 y	Qualitative study with focus group interviews Focus group with semi-structured interview on medical vocabulary	Many participants had inadequate health knowledge and insufficient access to health information Many were not aware of the definition and the importance of preventive care, cancer screening, mammography, Pap smears, hormone replacement therapy, mammography, and BSE Although all participants were insured, just 63% reported having a breast exam, Pap smear, or pelvic exam in the past year, and only 47.4% had received a mammogram Participants did not have sufficient knowledge on cardiovascular health screening, health risk assessments, and preventive measures Participants reported the absence of common language between them and their physicians, impacting their health knowledge Participants felt that they had reduced access to health information than the hearing population and had to be more dependent on peers to receive information
Terry et al.; 2016; Australia	To investigate health awareness and health information access among the Deaf	*n =* 40 (21 interview, 17 questionnaire, 2 Deaf service health providers) Questionnaire: 8 M, 9 F Interview: 8 M, 13 F Questionnaire: x- = 57.9 y, Range = 22 to 82 y, Interview: Age = Early 20s–80s	Mixed method approach Questionnaire and semi structured interview	The concept of health was not well understood, especially in relation to mental health, sexual health, and substance use 15 (88.2%) had never accessed services related to mental health, sexual health, or drug and alcohol support Besides the hospital and their own physicians, most were not aware about other health services in the community Sources of information for younger participants Parents School Friends Older participants usually had to learn through their personal experiences
Sexual and reproductive health literacy (*n =* 5)				
Francisqueti Marquete et al.; 2023; Brazil	To explore the knowledge and behaviors of the Deaf regarding sexually transmitted infections	*n =* 110 53 M, 57 F Age = 18–73 y Median = 32.0 y	Cross-sectional study Questionnaire on knowledge of sexually transmitted infection (STIs)	Most participants had limited knowledge about STIs; < 40% of correct answers regarding hepatitis, gonorrhea, and syphilis. Bimodal bilingual participants had more knowledge on STIs Bimodal bilingual participants were 4.29 times more likely to know that HIV can be transmitted through shared syringes (*p* = .0026) and 4.07 times more likely to know that condoms prevent hepatitis transmission (*p* = .0074) 44.9% reported no access to health guidelines. Being bimodal bilingual did not have significant effect on having access to health guidelines (*p* = .0778)
Mprah et al.^u^, 2017; Ghana	To explore knowledge of family planning practices among the Deaf	*n =* 178 (26 focus group, 152 survey) Focus group: seven male executives from Ghana National Association of the Deaf, 10 M, 9 F Survey: 87 M, 65 F Age 18–25: 92 26–35: 59 36–45: 9 46–55: 12 56 and above: 6	Mix-methods design Focus group and survey	Women in the focus group felt that Deaf people have insufficient knowledge on pregnancy All male executives in the focus group felt that the Deaf had no awareness about the adverse outcomes of unprotected sex Only 38.7% M and 31.6% F could answer correctly on pregnancy prevention practices Only 29.0% M and 30.8% F knew that a women could be pregnant even if she showered immediately after sexual intercourse Only 42.5% M and 43.1% F were aware that a single instance of unprotected sex could result in pregnancy 16% believed pregnancy can be prevented by avoiding blood transfusions and 9% by avoiding kissing Only 41.4% M and 46.2% F correctly identified that avoiding sex prevents pregnancy; some confused pregnancy prevention with STI/STD prevention
Spellun et al^v^, 2019; US	To compare HPV knowledge and risk perception of the Deaf and hearing	*n =* 354 (236 Deaf, 118 hearing) Gender = N/A Age = N/A	Cross-sectional study Health information national trends survey	58% of Deaf participants had heard of HPV versus 84% of hearing participants (*p* < .001) Deaf women significantly more likely to have heard of HPV versus Deaf men (*p* < .05) No significant differences in HPV awareness for different races, education levels, occupation, and health insurance (*p* > .05) Hearing participants significantly more likely to associate HPV with cervical cancer versus Deaf participants (*p* < .001) A significantly higher number of hearing respondents had heard of HPV vaccine (*p* < .001) and believed the effectiveness of HPV vaccine in prevention of cervical cancer versus Deaf respondents (*p* < .01) Hearing participants reported significantly better patient-centered communication (PCC) experiences versus Deaf participants (76 versus 62; *p* < .001)
Suariyani et al.; 2020; Indonesia	To explore reproductive health knowledge among the Deaf	*n =* 6 3 M, 3 F Age = 15–16 y	Qualitative study with interviews Semi-structured interview on reproductive health knowledge	Lack of reproductive health knowledge Preferred internet as source of reproductive health information as it was easier and quicker for them Parents were expected to provide reproductive health education, but cultural taboos and embarrassment prevented open discussions Suggested that the health information should be tailored to them such as adding sign language
Swartz DB; 1993; US	To obtain the knowledge of sex information among Deaf college freshmen	*n =* 38 Gender: N/A Age: N/A	Survey using the sex knowledge inventory (SKI) scale	Hearing subjects performed significantly better (x- correct: 80%, SD: 0.50) versus Deaf subjects (x- correct: 71.4%, SD: 0.07); *p* < .001
Cardiovascular literacy (*n =* 4)				
Haricharan et al.; 2017; South Africa	To evaluate the effectiveness of an SMS-based health promotion campaign in increasing Deaf individuals’ knowledge on hypertension	*n =* 41 25 M, 16 F x- = 45 y (± SD 12)	Pretest-posttest study Questionnaire on knowledge of hypertension and healthy living SMSs with medical information on hypertension and how to manage or avoid it through healthy living	Knowledge increased significantly from baseline to post-intervention during follow up (*p* = .0033) 6/19 questions showed a statistically significant increase in knowledge Most participants (80%) reported SMS to be effective in increasing their hypertension knowledge, and most (78%) found the SMS easy to comprehend 46% normally used SMS as a source of information, followed by written material (24%), internet (17%) and friends, family and colleagues (15%); 20% stated that they do not receive any health information Most effective in providing health information Deaf organizations (56%) SMSs (27%) Suggested making SMSs easier to understand by using simpler language, sign language structure, and adding pictures to support visual communication
Margellos-Anast et al.; 2006; US	To explore the cardiovascular disease knowledge among the Deaf patients	*n =* 203 93 M, 110 F x- = 44.8 y	Cross-sectional study Survey on cardiovascular knowledge	40.4% did not know any common heart attack symptoms; 62.6% did not know any stroke symptom 61% of participants stated they would dial 911 if they believed they were experiencing a heart attack or stroke versus hearing population (75%–79%) Among the 39% who said they would not call 911, most explained they would not think of it, considered it inaccessible for Deaf individuals, or lacked trust in its response. 32% could not list any risk factors for CV disease. Specific risk factors were more likely to be identified by those who had them A significant difference (*p* < .05) in mean number identified, median number identified and % of participants unable to correctly identify any risk factors between participants with different: Race or ethnicity Education level Income level Participants who were white, education level of high school or higher and income level of >$20,000 identified more symptoms versus participants who were black or other race or ethnicity, education < than high school and income ≤$20,000 (*p* < .05) No significant association for gender and age
McKee et al.; 2011; US	To identify the cardiovascular knowledge among Deaf ASL users.	*n =* 22 9 M, 13 F x- = 55 y, R = 41–69 y	Qualitative study with focus group Focus group with open-ended and semi structured questions	Good awareness on common heart disease symptoms, the health effect of smoking, the benefits of physical activities, and the benefit of decreasing salt intake and stress level Misinformation and knowledge gap on adverse effects of illegal drugs, anatomy of the brain and heart, and medications Reported language and communication barriers, decreasing their access to health information Sources of health information ASL-accessible workshops Captioned television shows ASL-accessible health videos and medical websites ASL fluent medical professionals The deaths of famous celebrities from diseases. Felt interpreters and HCPs who were ASL users could encourage and provide access to health information
Smith et al.; 2015; US	To explore the knowledge, access and barriers in accessing cardiovascular health information among the deaf adolescent	*n =* 20 11 M, 9 F Age = 14 to 19 y	Qualitative study with interviews Semi structured interview in focus group on cardiovascular knowledge	Knew the benefits of having a healthy diet and regularly engaging in physical activities, the importance of not smoking and common CVS disease symptoms; many unable to explain the causes of heart attack and stroke or describe cholesterol A few complained that the health information provided by their health education teachers were too brief and simple and did not help them much All reported lack of initiative by their teachers to teach them about their family medical history Many reported difficulty in understanding health terminology despite having higher English literacy, leading to reduced knowledge on more complex health issues There was a lack of accessible health information via printed and digital media Many had problems following spoken languages during family conversation, making it more difficult to access health information via informal learning Only limited opportunities for informal learning besides family
COVID-19 literacy (*n =* 4)				
Almusawi et al.; 2021; Kuwait and Saudi Arabia	To identify any differences in knowledge and practice regarding COVID-19 between the hearing and DHH	*n =* 110 (70 Hearing, 40 DHH) 37 M, 73 F ≤ 20 y: 9.09% 21–30 y: 46.36% 31–40 y: 24.55% 41–50 y: 15.45% 51–60 y: 4.55%	Cross-sectional design Questionnaire^z^ on Knowledge of COVID-19^n^	DHH had a lower score on 15/19 questions versus hearing Exclusive sign language users (*p* = .0018) and DHH with moderate and worse hearing-impaired levels (*p* = .0349) were predicted to score lower DHH participants performed better in understanding the use of facemasks (92.5%) versus hearing participants (81.43%) Age and education level were not associated with lower knowledge scores Source of DHH COVID-19 information Social media (65%) Official government websites (57.5%) Family and friends (47.5%) Sign language interpreters on TV (25%) Most hearing (84.3%) received their COVID-19 information from official government websites
James et al.; 2022; US	To explore health literacy and access to COVID-19 information of DHH	*n =* 417 417 F x- = 35.54 y (± SD 5.77), Age = 22–49 y	Cross-sectional study Single item measurement: “How confident are you filling out medical forms by yourself?”	17.1% (72 participants) had inadequate health literacy 22.1% (93 participants) of the respondents faced challenges when accessing COVID-19 information Inadequate health literacy was strongly associated with information barriers (adjusted OR = 2.21) Participants with lower educational level, inadequate health literacy and Hispanic participants were more likely to have barriers accessing COVID-19 information 18 respondents describe difficulties in Accessing COVID-19 information from public broadcasts Acquiring information on testing and vaccinations Identifying trustworthy COVID-19 information Participants reported inadequate reliable captioning on news channels and problems with ASL interpreters being excluded or not fully visible on screen during public emergency broadcasts
Jnr et al.; 2023; Ghana	To determine the KAP of COVID-19 among Deaf individuals	*n =* 144 68 M, 76 F; x- = 36 y (± SD 12.84)	Cross-sectional study KAP COVID-19 instrument (questionnaire)^o^	67% of the participants answered 8/12 questions wrong Key gaps were seen in knowledge of the cause, its viral nature, incubation, and diagnosis. For instance, only 13.9% knew COVID-19 is viral, 95.8% were unaware it is RNA-based, and 97.2% did not know it is called SARS-CoV-2 Deaf persons showed good attitudes and adherence to preventive practices but lacked knowledge about the science of COVID-19 Deaf persons consistently followed preventive measures (masks, no contact, ventilation, handwashing, sanitizers) and sometimes practiced (distancing, respiratory hygiene, avoiding face-touching and staying home when ill)
Paludneviciene et al.; 2021; US	To explore COVID-19 knowledge and risk perception among DHH	*n =* 475 203 M, 256 F, 16 non-binaries Age = 18–88 y	Cross-sectional study Survey on COVID-19 knowledge	88% reported they were well-informed about physical distancing 72% reported that they understood the impact of physical distancing in preventing the spread of COVID-19 70% reported the understanding the contagiousness of infected asymptomatic individuals Participants with a college degree were nearly five times more likely to know about the contagiousness of infected asymptomatic individuals than participants with an education level of high school or lesser. (adjusted odds ratio = 4.67, 95% CI 2.22–9.80)
Oral health literacy (*n =* 4)				
Kumar et al.; 2022; India	To explore the effect of OHE using VPR technique versus sign language, on oral health knowledge among the Deaf adolescents	*n =* 80 28 M, 52 F Experimental: x- = 13.22 y (± SD 0.74) Control: x- = 13.76 y (± SD 0.91)	Randomized controlled trial Questionnaire on oral hygiene knowledge Experimental: VPR technique (animated video) Control: OHE in sign language	The VPR experimental group scored (5.71 ± 1.64) significantly higher post-intervention versus sign language control group (3.54 ± 1.71); and at the 16-week follow-up versus control group (*p* < .001) % of correct scores significantly higher in post-intervention versus baseline (*p* < .05) for both groups Significant improvement in oral hygiene practices between both groups observed post-intervention Both groups showed a statistically significant reduction in gingival and plaque scores from baseline to the 16-week interval (*p* < .001)
Oredugba et al.; 2004; Nigeria	To explore oral health knowledge and practices among Deaf adolescents	*n =* 50 26 M, 24 F x- = 13.3 y (± SD 2.8)	Cross-sectional study Questionnaire on oral health knowledge	Most participants knew the causes of bleeding gums (72%) and the function of teeth (82%) Only a minority of participants knew the causes of tooth decay (20%) and the purpose of toothbrushing (14%)
Santhosh et al., 2025; India	To assess the effect of the JPVR method in improving Deaf adolescents’ toothbrushing knowledge, practices, and their oral health	*n =* 90 30 M, 60 F Age between 12 and 15 years old	Single- blind randomized controlled trial Questionnaire on knowledge and practices on toothbrushing Intervention group: OHE using sign language followed by JPVR; control group: OHE using sign language	Both groups showed a significant improvement in knowledge and practice scores at 3 months versus baseline (*p* = .001) The JPVR group demonstrated a significantly greater increase in knowledge versus control group after 3 months (*p* = .001) The JPVR group showed a significant greater improvement in practice scores versus control group at 3 months (*p* = .001) At the end of 3 months, the JPVR group had a significantly lower mean plaque score versus control group (*p* = .001)
Soesilaningtyas et al. 2018; Indonesia	To evaluate a story book with pictures as an intervention in improving oral health knowledge of deaf students	*n =* 36 15 M, 21 F 9–10 y: 19.4% 11–12 y: 41.7% 13–14 y: 38.9%	Pretest-posttest study design; Questionnaire on oral health knowledge; Experimental: OHE via story book with picture, Control: lecture with sign language	Knowledge level before any interventions Good knowledge: 5.6% Sufficient knowledge: 55.6% Poor knowledge: 38.8% There was a significant improvement in knowledge scores for both groups between post-test and pre-test results (*p* < .05) The experimental group had a significant increase in their knowledge from a mean baseline score of 53.89 ± 20.04 out of a total 100 points to a post-intervention score of 82.78 ± 19.34 (*p* = .000). The experimental group had a higher positive change in their post-intervention knowledge score (28.89 ± 13.67) than the control group (12.22 ± 9.2) (*p* < .05)
Women’s Health Literacy (*n =* 4)				
Arunachalam et al.; 2020; India	To explore menstrual hygiene knowledge and practices of Deaf and mute adolescent girls	*n =* 22 Age = N/A	Cross-sectional design; Questionnaire on menstrual hygiene^p^	Adequate knowledge: 27.3% Moderately adequate knowledge: 45.5% Inadequate of knowledge: 27.3% > 80% demonstrated good menstrual hygiene practices, including daily bathing, frequent genital cleaning, use of sanitary pads, and safe disposal methods
Esmeray & Yanikkerem, 2022; Turkey	To evaluate how educational interventions influence deaf women’s knowledge and attitudes regarding Pap smear tests	*n =* 156 Control: x- = 53.0 y (± SD 8.8) Intervention: x- = 47.2 y (± SD 11.1)	Controlled trial study with longitudinal design Cervical cancer (CC) and pap smear test (PST) Information Assessment Form^q^ Experimental: education about cervical cancer and Pap smear test via face-to-face interviews using Turkish sign language Control: training from a routine health institution.	Pre-intervention knowledge of CC and PST very limited, with mean scores of 1.1 (control) and 0.6 (intervention) out of 10. No participants knew when a Pap smear should be done Participants’ score improved significantly from pre intervention (0.6 ± 1.6 out of 10) to post-intervention (9.2 ± 1.4) (*p* < .001) Awareness of the location of the cervix increased from only 7.7% to 97.4% after education (*p* < .001) Awareness of the purpose of a PST increased from 11.5% to 98.7% (*p* < .001) After 3 months of education, 29.5% of women in the intervention group had a PST versus only 1.3% in the control group (*p* < .0001)
KocakAkgun et al.; 2022; Turkey	To evaluate a video on BSE skills for Deaf women	*n =* 60 (30 experimental, 30 control) Experimental: x- = 61.7 y (± SD 9.5) Control: x- = 50.0 y (± SD 13.2)	Pretest-posttest Checklist for BSE^r^ Experimental: BSE training video including sign language Control: BSE training video not including sign language	Women who watched the video with sign language (Group A) demonstrated significantly better BSE skills versus those who watched the video without sign language (Group B) 93.3% of Group A “met expectations” in identifying differences in breast shape, size, or skin changes, versus only 36.7% in Group B (*p* < .05) 93.3% of Group A used the pads of their fingers correctly versus 3.3% in Group B (*p* < .05) 96.7% of Group A palpated the axilla correctly versus 10% in Group B (*p* < .05)
Wollin et al.; 2003; Australia	To explore knowledge of mammograms and pap smears among Deaf women	*n =* 13 Middle-aged or older	Qualitative study with interviews Face-to-face interview on mammograms and Pap smear knowledge	11 participants were familiar with mammograms, but their knowledge was often incomplete or inaccurate Nine correctly identified mammograms as a breast cancer screening tool Seven knew the recommended two-year interval, whereas five did not know the correct frequency All participants knew of Pap smears, but nearly half were unsure or misinformed about their purpose Nine participants correctly reported that Pap smears should be performed every two years, while four did not know the recommended frequency
Smoking Literacy (*n =* 1)				
Berman et al., 2011; US	To develop and evaluate a tobacco prevention curriculum designed for DHH students.	*n =* 618; 308 M, 310 F; Intervention school 1: x- = 16.0 y (± SD 2.2) Intervention school 2: x- = 15.1 y (± SD 2.0) Control school 1: x- = 15.5 y (± SD 1.9) Control school 2: x- = 15.6 y (± SD 1.8)	Pretest-posttest study California student tobacco survey^s^ “Hands Off Tobacco! An Anti-Tobacco Program for Deaf Youth”, a school-based curriculum	The baseline range for x- knowledge was 60 to 76 points out of 100 Scores in Intervention school 2 increased by 11.4 points (*p* < .001). No significant changes in points for other schools There was also significant improvement in x- anti-tobacco attitude scores and mean tobacco education exposure scores in both intervention schools (*p* < .001)
Concussion literacy (*n =* 1)				
Brancaleone et al.; 2022; US	To study knowledge and attitudes toward concussion in DHH athletes.	*n =* 53; 28 M, 25 F Age = N/A	Cross-sectional study Rosenbaum concussion knowledge and attitudes survey (RoCKAS^)t^	The mean CKI score was 16.25 ± 3.83 The mean CAI score was 58.04 ± 6.44 Athletes who identified as White had significantly higher concussion knowledge scores (17.53 ± 3.17 out of 25) versus People of Color (14.28 ± 3.98) (*p* < .01) Gender and primary mode of communication (American Sign Language versus English) had no significant effect on knowledge level
Dementia literacy (*n =* 1)				
Ferguson-Coleman et al. 2014; UK	To examine the knowledge of Deaf individuals regarding dementia and their access to services	*n =* 26 14 M, 12 F; Age = 18–60 y	Qualitative study with focus group interviews Focus group interviews to evaluate knowledge about dementia and access to services to gain knowledge	Knowledge level depended on personal experiences with family Most knew there was no cure for dementia and believed there were no prevention strategies Participants misunderstood dementia, associating it with mental illness, unusual behavior, or appearance Participants mentioned that complexity of information versus unfamiliar contents influence the understanding of written information. Many preferred a peer-to-peer support model, with Deaf individuals leading the support groups within the Deaf community
Senescence disorder literacy (*n =* 1)				
Hart et al.; 2017; US	To explore the knowledge of age-related disorders among the Deaf	*n =* 27 14 M, 13 F Male age: x- = 73.85 y (± SD 1.9) Female age; x- = 64.92 y (±SD 2.8)	Post-positivism quantitative research Modified version of the REALM	Inadequate health literacy: 7.4% (*n =* 2) Semi conversant: 48.1% (*n =* 13) Sufficient health literacy: 18.5% (*n =* 5) Women had significantly higher scores versus males on Medical Conditions, Medical Procedures, and Medical Instructions
Mental health literacy (*n =* 1)				
Opoku et al., 2024; Ghana	To investigate Deaf individuals’ knowledge of mental health and their access to related services	Phase 1 of study (Questionnaire), *n =* 284 Phase 1: 110 M, 164 F Phase 2 of study (interview), *n =* 40 20 years old and above	Mixed-methods design Questionnaire and interview	Awareness: Schizophrenia: 76% Depression: 54% All conditions: ≥50% awareness Attributed mental health conditions to different causes e.g., stressful life events (50%–60%), genetic factors (45%–50%), curses (44%–53%), and witchcraft (33%–42%), with stress being the most identified cause overall Friends (55%) were the most common source of mental health information, followed by media (50%), social media (43%), and school (38%), with parents (37%) being the least cited source 70% were aware of health facilities offering mental health services During interview, some participants believed that mental illness was commonly linked to drug use, old age, or congenital factors; some believed anyone could be affected Most believed deafness can lead to mental health issues Commonly accessed mental health information through the Internet and social media, which they found most accessible due to smartphone use Teachers were also helpful as they had basic sign language skills Television and family were the least accessible sources as television lacked interpreters or had poorly visible signing, and family members struggled to explain information
Premarital screening literacy (*n =* 1)				
Zaien et al.; 2022; Saudi Arabia	To explore the knowledge of premarital screening and genetic counseling (PMSGC) among DHH women	*n =* 67 x- = 23.64 y (± SD 4.04)	Cross-sectional study Electronic questionnaire on PMSGC knowledge	Most participants responded incorrectly to the majority of PMSGC questions, particularly the following: Meaning of premarital screening (70.1%) Meaning of genetic counseling (70.1%) Infectious blood-borne disease detected by PMSGC (76.1%) Suitable period for PMSGC (70.1%) Reliability of a healthy marriage certificate (80.6%). Education significantly associated with PMSGC knowledge (*p* = .019), where university students scored significantly higher versus secondary school students (*p* = .000) PMSGC knowledge significantly associated with age (*p* = .007) and income (*p* = .015), with higher knowledge observed in participants aged ≥20 or with lower monthly income. No association observed between degree of hearing and PMSGC knowledge

^a^US: United States; UK: United Kingdom; AIDS: acquired immunodeficiency syndrome; *n*: total number of participants.

N/A: not available; M: males; F: females; HIV: human immunodeficiency virus; y: years old; x-: mean; SD: standard deviation; DHH: deaf and hard-of-hearing; ASL: American sign language; COVID-19: corona virus disease; KAP: knowledge, attitudes, and perceptions; VPR: visual performance reinforcement; OHE: oral health education; HPV: human papillomavirus; CKI: concussion knowledge index; CAI: concussion attitudes index; REALM: rapid estimate of health literacy in medicine; PMSGC: premarital screening and genetic counseling; JPVR: jigsaw puzzle-assisted visual reinforcement; BSE: breast self-examination.

b Quiz on cervical cancer knowledge: ranges from 0 to 22. Poor knowledge: < 11, fair: 11–16.5, good: 16.6–22.

c Skin cancer knowledge questionnaire (SCKQ): 11 multiple-choice questions; 29 true/false. Ranges from 0 to 40 points.

d Korean Health Literacy Assessment Tool-2 (KHLAT-2): 66 words with the options of “knowing exactly” or “not knowing exactly”, ranges from 0 to 66 points. 0–18 points: 0–3rd grade; 19–44 points: 4–6th grade; 45–60 points: 7–8th grade; 61–66 points: > 9th grade.

e Short Form of the Korean Functional Health Literacy (S-KFHLT): four questions each on the two domains: Numeracy and reading comprehension domains, ranges from 0 to 8 points, higher points indicate higher functional health literacy.

f Digital Health Technology Literacy Assessment Questionnaire (DHTL-AQ): Ranges from 0 to 34 points, higher points indicate higher internet health literacy.

g Turkish Health Literacy Scale (TSY-32): Ranges from 0 to 50 points. 0–25 = insufficient, > 25–33 = problematic-limited, > 33–42 = sufficient, > 42–50 = excellent.

h S-TOFLA: Short Test of Functional Health Literacy in Adults. Measures capability to read and understand health information in English.

i Interactive health literacy questionnaire; two questions measuring interactive health literacy. Rated from 1(never) to 5 (always).

j Interactive health literacy: Qualitative response. Response was given a score from 0 to 4 points.

k Newest vital sign: 66 questions on nutrition label. 0–1 correct = inadequate health literacy; 2–4 correct = at risk of limited health literacy; 5–6 correct = adequate health literacy.

l Wagner’s heart disease fact questionnaire: 25 true or false questions.

m Only 20 participants in the focus group responded for the age section.

n Questionnaire on knowledge of COVID-19: 35 items on demographics, knowledge, sources of information and opinions on whether they were well-informed and needed more advice, and on the health services availability.

^0^KAP COVID-19 instrument (questionnaire): assess knowledge (12 questions), attitude (6 questions) and adherence to COVID-19 preventive practices (9 questions).

p Questionnaire on menstrual hygiene: ≥67% = acceptable knowledge; 51%–67% = moderately appropriate; ≤ 50% = inadequate.

q Cervical cancer and pap smear test information assessment form: seven questions, one point each for correct answers for Questions 1, 2, 4, 5, and 6, three points for Question 3, two points for Question 7. Scores ranges from 0 to 10 points.

r Checklist for BSE: nine tasks on breast examination skills. Grades given accordingly: “Meets expectations”: performed completely and correctly; “Needs improvement”: performed incompletely or incorrectly.

s California student tobacco survey: 90-item student survey on demographics, knowledge, attitudes and practices, and exposure; 12 items on tobacco-related knowledge, score was on a scale of 0–100, higher scores mean better knowledge.

t Rosenbaum concussion knowledge and attitudes survey (RoCKAS): 55 items with two subscales, the CKI and the CAI. CKI score on a scale of 0 to 25; higher scores indicate higher knowledge

u The population reported in the two papers by Mprah et al. were similar.

v Due to the ambiguity in the demographic data, these numbers were not included in the total numbers reported above.

w The Deaf sample used was the same as the study by Choe et al.

x Due to the ambiguity in the demographic data, these numbers are not included in the total numbers reported above.

y The gender for two participants were not reported.

### Key findings

Four key themes were identified from the 57 included studies: (1) health literacy of Deaf people, (2) factors affecting the health literacy of Deaf people, (3) source of health information, and (4) impact of interventions on health literacy levels.

#### Theme 1: health literacy of Deaf people

The overall trend of the studies showed low literacy levels in all diseases, save for studies by Doyle (1995) and Flowers (1998) on HIV/AIDS literacy, Kushalnagar et al. (2018) on general health literacy, and Paludneviciene et al. (2021) on COVID-19 literacy.

Of the 12 studies reporting on HIV/AIDS, 10 studies described Deaf participants as having inadequate health literacy on HIV/AIDS (Baker-Duncan et al., 1997; Bat-Chava et al., 2005; Bisol et al., 2008; De Andrade & Baloyi, 2010; Doyle, 1995; Flowers, 1998; Goldstein et al., 2010; Groce et al., 2006; Groce et al., 2007; Heuttel & Rothstein, 2001; Mprah, 2013; Woodroffe et al., 1998). In the article by Bisol et al. (2008), Deaf students had significantly poorer HIV knowledge than hearing participants (*p* < .001), with less than half (47%) of Deaf students answering at least seven out of 16 questions correctly. There was also a lack of awareness about the vertical transmission of HIV (Groce et al., 2007), misconceptions that kissing (Groce et al., 2007) and sharing marijuana (Bat-Chava et al., 2005) could result in HIV transmission, and poor awareness of the association between multiple partners and the risk factor of contracting HIV (De Andrade & Baloyi, 2010; Woodroffe et al., 1998). In contrast, the study by Doyle (1995) found that Deaf participants had relatively high AIDS knowledge scores with a mean of 81%, while Flowers (1998) reported that close to 80% of participants had adequate HIV/AIDS knowledge, with more than 90% understanding vertical transmission of HIV.

Seven out of the 12 studies on cancer literacy reported low knowledge among Deaf individuals (El Sayed et al., 2022; Hickey et al., 2013; Jensen et al., 2013; Orsi et al., 2007; Sacks et al., 2013; Sadler et al., 2001; Yao et al., 2012). Deaf participants had lower knowledge of breast cancer, with Hickey et al. (2013) showing a lack of knowledge especially with regard to the age when one should start breast examinations, either by oneself or with a HCP, while in the Sadler et al. (2001) study, more than 50% of Deaf women had “no knowledge” of testicular and colorectal cancer, more than 40% had “no knowledge” of prostate cancer, and more than 30% had “no knowledge” of ovarian and cervical cancer. No Deaf participants in the El Sayed et al. (2022) study had “good knowledge” of cervical cancer, with 45% having “poor knowledge”, and 55% having “fair knowledge”; while in the cross-sectional study by Orsi et al. (2007), less than half of the participants (48%) could define a Pap smear.

Four out of the five studies that focused on general health literacy found a pattern of poor literacy (Choi et al., 2023; Gur et al., 2020; McKee et al., 2015; Terry et al., 2016), while Kushalnagar et al. (2018) reported that most Deaf participants had adequate functional health literacy. Choi et al. (2023) presented data showing the inadequacy of health literacy in Deaf participants across all three domains of linguistic, functional, and internet health literacy. Moreover, Gur et al. (2020) noted that only 10% (*n =* 9) of Deaf participants had adequate health literacy, and nearly half of them (43%, *n =* 38) reported that they were unable to adhere to their prescribed medications because they could not comprehend the usage directions, as a repercussion of their poor health literacy. Three out of the four papers on COVID-19 literacy reported lower literacy levels (Almusawi et al., 2021; James et al., 2022; Jnr et al., 2023). In two out of the four papers on OHE, Deaf individuals had poorer literacy levels with less than 6% having “good knowledge” in the Soesilaningtyas et al. study (2018), while less than 15% understood the purpose of brushing teeth and less than 25% knew the causes of tooth decay in the Oredugba (2004) study. The latter study did report “good knowledge” of the causes of bleeding gums and teeth function (Oredugba, 2004), while Kumar et al. (2022) found equal knowledge compared to the hearing population.

With regard to CVD literacy, more than 60% of Deaf participants could not list the symptoms of a stroke, while approximately 40% did not know common symptoms for a heart attack in the Margellos-Anast et al. (2006) study. In the McKee et al. (2011) study, however, Deaf individuals had better awareness of the common symptoms of heart disease, but were confused about medication and lacked knowledge about the impact of illicit drugs on the heart. Similarly, the study involving Deaf adolescents by Smith et al. (2015) found that while they possessed good knowledge on the basic CVD symptoms, most lacked knowledge about what occurs during a heart attack and stroke, and could not accurately describe what cholesterol was. Studies also found that Deaf people had poor knowledge on HPV (Spellun et al., 2019), pregnancy (Mprah et al., 2017), reproductive health (Suariyani et al., 2020), and sexually transmitted diseases such as gonorrhea and syphilis (Francisqueti Marquete et al., 2023). Deaf women also had poor knowledge about Pap smears (Esmeray & Yanikkerem, 2022; Wollin & Elder, 2003), mammograms (Wollin & Elder, 2003), and menstrual health hygiene, with less than 30% having adequate knowledge of the latter (Arunachalam et al., 2020).

Subtheme 1: Health literacy of the Deaf community compared to the hearing population. Eleven studies compared Deaf peoples’ health literacy to the hearing population, with all studies showing that Deaf participants had poorer health literacy than hearing participants. Three studies found that Deaf individuals had lower knowledge about cancer than the hearing (Jensen et al., 2013; Sacks et al., 2013; Yao et al., 2012), where they had significantly lower literacy levels on general health (Jensen et al., 2013; Sacks et al., 2013; Yao et al., 2012), ovarian (Jensen et al., 2013), testicular (Sacks et al., 2013), cervical (Yao et al., 2012), and total cancer (Jensen et al., 2013; Sacks et al., 2013; Yao et al., 2012) compared to typically hearing people. Five papers found lower HIV/AIDS literacy levels among Deaf participants (Bisol et al., 2008; Groce et al., 2006; Groce et al., 2007; Heuttel & Rothstein, 2001; Woodroffe et al., 1998). For instance 91%(*n =* 42) of the hearing participants in the study by Heuttel & Rothstein (2001), were able to answer 90% of an HIV/AIDS questionnaire correctly, in contrast to only 41% (*n =* 14) of Deaf subjects. Regarding knowledge of COVID-19, a 2021 paper by Almusawi et al. (2021) reported that Deaf participants scored less on 15 out of 19 questions than hearing participants. McKee et al. (2015) found that nearly half of Deaf participants demonstrated inadequate health literacy, with Deaf individuals significantly more likely than typically hearing individuals to fall into this category, while Swartz (1993) found that Deaf freshmen had poorer knowledge on sex compared to hearing students.

#### Theme 2: factors affecting the health literacy of Deaf people

Fifteen studies highlighted demographic factors that were associated with the health literacy of Deaf people. Factors that were commonly explored were age, gender, race or ethnicity, education level, and income level. Three studies reported age as a significant factor affecting health literacy, with older participants being more likely to have better knowledge (Flowers, 1998; Gur et al., 2020; Zaien et al., 2022). This finding was different from four other studies which did not find any associations between age and health literacy levels (Almusawi et al., 2021; Hickey et al., 2013; Kaskowitz et al., 2006; Margellos-Anast et al., 2006). Nine studies reported education level as a factor, with most studies (*n =* 7) (Goldstein et al., 2010; Hickey et al., 2013; Kaskowitz et al., 2006; Margellos-Anast et al., 2006; McKee et al., 2015; Paludneviciene et al., 2021; Zaien et al., 2022) finding that higher education was positively associated with higher health literacy. The other two studies showed no significant association (Almusawi et al., 2021; Spellun et al., 2019). In three studies, White participants tended to have better health knowledge (Brancaleone & Shingles, 2022; Goldstein et al., 2010; Margellos-Anast et al., 2006). However, three other studies found that neither race nor ethnicity had any role in affecting health literacy levels (Doyle, 1995; McKee et al., 2015; Spellun et al., 2019).

Two studies reported that female participants scored higher than males on certain components of the knowledge scale (Doyle, 1995; Hart, 2017),whereas another study found lower health literacy among females participants (Gur et al., 2020). Nevertheless, several studies did not discover any notable association between gender and health literacy levels among Deaf participants (Brancaleone & Shingles, 2022; Flowers, 1998; Margellos-Anast et al., 2006; McKee et al., 2015). Studies found that participants with higher-income and better economic status had higher health literacy (Gur et al., 2020; Margellos-Anast et al., 2006; McKee et al., 2015), save for one study by Zaien et al. (2022) which found higher literacy levels in those with a lower monthly income.

#### Theme 3: source of health information

Nineteen papers identified the primary sources of information used by the Deaf community to obtain their health information. Various responses, including family, friends, school, the Internet, printed materials, and community or health services were reported. Family, friends, and schools were mentioned in 10 studies as a common source of health information (Almusawi et al., 2021; Bat-Chava et al., 2005; Doyle, 1995; Folkins et al., 2005; Goldstein et al., 2010; Heuttel & Rothstein, 2001; Opoku et al., 2024; Sadler et al., 2001; Smith et al., 2015; Terry et al., 2016). It was, however, observed that the primary source of information that Deaf people used varied according to age and was mainly disease specific.

In the context of acquiring information about COVID-19, Deaf participants relied more on social media, whereas hearing individuals’ main source of information was from official government websites (Almusawi et al., 2021). Deaf individuals did not have access to reliable sources, and none of the sources could explain “coronaviruses” to them (James et al., 2022). Three out of the six studies (Bat-Chava et al., 2005; Doyle, 1995; Goldstein et al., 2010; Groce et al., 2006; Groce et al., 2007; Heuttel & Rothstein, 2001) that focused on HIV/AIDS concluded that school (Bat-Chava et al., 2005; Goldstein et al., 2010) and friends (Doyle, 1995) were the two primary information sources for acquiring HIV/AIDS-related health information. However, in contrast with the hearing population, Deaf participants depended heavily on their family and friends as a source of HIV information (Heuttel & Rothstein, 2001). In a 2006 study involving Deaf individuals in Swaziland, Groce et al. (2006) found that 94% (*n =* 94) of hearing participants could obtain health information from hospitals, unlike Deaf individuals (24%, *n =* 22), with the latter reporting printed materials (posters) and community services like disabled people’s organizations (DPOs) as the most commonly reported sources of information. A 2007 study in Nigeria by the same author found that Deaf participants had significantly less (*p* < .05) information accessibility from television and written sources like posters and newspapers than the hearing population (Groce et al., 2007).

With regard to cancer health literacy, two articles documented HCPs as the primary source of cancer information for Deaf participants (Orsi et al., 2007; Sadler et al., 2001). Other sources were printed materials and the internet (Kaskowitz et al., 2006; Sadler et al., 2001). In the Haricharan et al. (2017) study, more than 50% of Deaf respondents stated that deaf organizations are their primary source to obtain hypertension-related information. Compared to younger Deaf individuals, personal experience was the primary health information source identified in older Deaf participants (Terry et al., 2016). Deaf adolescents expressed that family was not an adequate source to obtain information on reproductive health as they were embarrassed, hence most of them turned to the Internet (Suariyani et al., 2020).

#### Theme 4: impact of interventions on health literacy levels

Seventeen papers assessed the impact of educational interventions on the health literacy levels of Deaf people related to specific diseases: 10 on cancer (Choe et al., 2009; El Sayed et al., 2022; Engelberg et al., 2019; Folkins et al., 2005; Harry et al., 2012; Hickey et al., 2013; Jensen et al., 2013; Kaskowitz et al., 2006; Sacks et al., 2013; Sadler et al., 2001), three on OHE (Kumar et al., 2022; Santhosh et al., 2025; Soesilaningtyas et al., 2018), and one each on smoking (Berman et al., 2011), hypertension (Haricharan et al., 2017), breast self-examination (Kocak Akgun & Ildan Calim, 2022), and Pap smear (Esmeray & Yanikkerem, 2022). The types of interventions used were varied—four studies used graphically-enhanced videos in sign language: Choe et al. focused on cervical cancer (Choe et al., 2009); Hickey et al. (2013) used pictorial explanations and summary graphics to increase breast cancer knowledge; while videos by Jensen et al. and Sacks et al. on ovarian (Jensen et al., 2013) and prostate and testicular (Sacks et al., 2013) cancer knowledge, respectively, included optional open-captions with English voice-overs. Two studies on OHE used visual performance reinforcement (VPR), with Santhosh et al. (2025) employing jigsaw puzzles, while Kumar et al. (2022) supplemented this with a demonstration of brushing techniques on a dental model. One study used a story book with pictures in sign language to increase OHE (Soesilaningtyas et al., 2018), Haricharan et al. (2017) used Short Message Service (SMS) to increase knowledge on hypertension, three studies employed educational videos in sign language to increase knowledge on prostate and testicular cancer (Folkins et al., 2005), skin cancer (Harry et al., 2012), and BSE (Kocak Akgun & Ildan Calim, 2022); while Engelberg et al. (2019) paired humor with health messages to increase knowledge on cancer.

The “Hands Off Tobacco! An Anti-Tobacco Program for Deaf Youth” curriculum by Berman et al. (2011) emphasized hands-on activities, graphic designs and visual elements, and used interactive teaching strategies such as role-play and art therapy; while Esmeray et al. (2022) employed face to face education using sign language along with a video and brochure to increase cervical cancer literacy. Three studies used PowerPoint or presentation slides in combination with sign language. Specifically, Kaskowitz et al. (2006) utilized slides to increase health literacy on prostate cancer, Sadler et al. (2001) incorporated slides along with visual displays and teaching aids to enhance knowledge of breast cancer, and El Sayed et al. (2022) employed presentations based on the Health Belief Model. The intervention also involved the use of colored papers and pens, audiovisuals, and different instructional methods such as lectures, group discussions, brainstorming, and concept mapping.

All interventions used sign language with the exception of the study by Haricharan et al. (2017) where English SMSes were used, and the Berman et al. (2011) study which did not specify if sign language was used in the Tobacco program. All studies specifically stated that the interventions were developed either in collaboration with Deaf people or based on feedback received from Deaf individuals, except for six studies (El Sayed et al., 2022; Esmeray & Yanikkerem, 2022; Kumar et al., 2022; Sacks et al., 2013; Santhosh et al., 2025; Soesilaningtyas et al., 2018). Repetition of content was utilized in four studies—the Berman et al. (2011) study on tobacco use which repeated basic concepts over seven classes; the Hickey et al. (2013) study on breast cancer which uploaded content to the internet to allow for repeated viewings; the study on OHE by Kumar et al. (2022) where periodic enforcement of content was provided at 1-, 4-, and 8-week intervals; and the study by Santosh et al. (2025) where participants were provided with visual aid stickers that they were asked to affix to their daily dairies to serve as visual reinforcements of the information acquired.

All programs/educational interventions resulted in significant increases in knowledge; however, only five studies mentioned retention of change in knowledge after a certain timepoint. The increase in knowledge on cancer was retained at 1-week in the Engelberg et al. (2019) study, while the significant increase in prostate, cervical, skin, and prostate and testicular cancer knowledge was retained after two months in the Kaskowitz et al. (2006), Choe et al. (2009), Harry et al. (2012), and Folkins et al. (2005) studies, respectively, although in the latter study this was slightly diminished. Repetition of material was not done in any of these five studies (Choe et al., 2009; Engelberg et al., 2019; Kaskowitz et al., 2006) (Folkins et al., 2005; Harry et al., 2012).

## Discussion

This scoping review mapped the existing literature on health literacy among Deaf populations and found four overarching themes: uneven health literacy levels, reliance on informal information sources, structural and communication barriers, and the need for accessible digital health solutions. Factors such as education level, income level, and mode of communication were associated with the health literacy of Deaf people. The review also found that educational programs employing various modalities such as graphically-enhanced videos in sign language, mobile health resources, and community-based education programs, were effective in increasing health literacy levels. The included studies varied widely in design and population characteristics. While most focused on adults, fewer studies examined children and young people. Several studies were excluded due to imprecise population definitions, especially as it pertained to specifying sign language usage. This is discussed further in the Limitations’ section.

### Health literacy of the Deaf community

Deaf individuals have been shown to have a similar trend of having unprotected sexual activity as the hearing population, leading to them have similar risks of contracting HIV (Bisol et al., 2008; Doyle, 1995; Taegtmeyer et al., 2009). However, some studies have reported higher risks in Deaf people (Hanass-Hancock & Satande, 2010; Margot et al., 2010) and disabled people (UNAIDS, 2017), particularly those in sub-Saharan African countries. We use the term “disabled” here as many organizations still follow a medical model of disability. As such Deaf individuals are still classified as disabled, even though most of them do not think of themselves as being disabled according to the medical model of deafness, but instead consider themselves to be disabled due to their environment, subscribing to the Social Model of Disability (Lane, 2002; Macdonald, 2009; Marks, 1997; Stebnicki & Coeling, 1999). As noted by Marks “Deafness becomes disabling in a community which does not recognise signing.” Despite facing comparable risks of HIV exposure, the lower knowledge of HIV, particularly the modes of transmission, may have led Deaf individuals to not perceive themselves to be susceptible to HIV and therefore may not consistently engage in preventative practices (Taegtmeyer et al., 2009). Indeed, an association was found between low health literacy regarding HIV consequences and involvement in unsafe sexual practices among the hearing population (Mmbaga et al., 2008). This is worrying given that in 2024, an estimated 40 million people were living with HIV, and while data shows a downward trend (World Health Organisation, 2025), Deaf people are still vulnerable due to limited access to information.

There is also a lack of awareness of preventative screenings among Deaf individuals which has led to delayed diagnosis and poorer prognosis due to delayed treatment, a finding highlighted in a retrospective analysis of the records of more than 4,000 Deaf patients in France, where it was found that 64% of the participants were diagnosed at advanced stages of cancer compared with the hearing population, which was only 13% (Druel et al., 2018). Similarly, our review also found poor oral health knowledge among Deaf participants. Poor oral health not only leads to poorer quality of life and increases the risk of oral cancer, diabetes, and atherosclerotic disease (Kane, 2017; Peres et al., 2019), but also carries with it a significant economic burden, ranking third behind diabetes and CVD at €80billion in terms of expenditure on diseases in European Union countries (Peres et al., 2019).

According to the WHO report on Global Health Estimates, ischemic heart disease is the leading cause of death, increasing by 2.7 million to 9 million deaths in 2021. This was followed by stroke (World Health Organisation, 2021). Studies have also shown that hearing loss posed a risk of increasing the CVD mortality rate in the Deaf community (Druel et al., 2018). However, despite having twice the risk of acquiring CVD, Deaf individuals were about half as likely to report a diagnosis compared to their hearing counterparts (SIGNHEALTH, 2014) and did not have proper medical prescriptions, mainly attributed to communication barriers (Gur et al., 2020). This observation is complemented by our findings which show that the majority of Deaf participants had poor CVD health literacy., which has been shown to lead to increased hospitalization and mortality, and poor adherence thereby exacerbating CVD outcomes among an already vulnerable population (Berkman et al., 2011; Williamson et al., 2025).

Other noncommunicable diseases such as Alzheimer’s Disease are also on the rise, making it to the top 10 global causes of death in 2021 (World Health Organisation, 2021). A meta-analysis found that hearing loss was significantly correlated with a higher risk of having dementia (Liang et al., 2021). However, Deaf people have poor knowledge of dementia, which contributes to a delay in help-seeking, missed opportunities for early diagnosis, and reduced access to timely support and care (Livingston et al., 2024), aligning with a study by Harris et al. that found that linguistic minorities such as Deaf individuals who suffered from dementia were often left undiagnosed for a prolonged period of time compared to the hearing population (Harris et al., 2021). This ultimately results in worsening disease outcomes and a burden on families, society, and the healthcare system (Livingston et al., 2024).

### Factors affecting the health literacy of Deaf people

This review highlighted that Deaf individuals with higher education levels having higher health literacy levels, similar to studies conducted among the hearing population (Jansen et al., 2018; Sudhakar et al., 2020). This association corresponds with the findings by McKee et al. (2014) that showed that individuals with lower education had a higher risk of CVD. The higher health literacy levels of Deaf participants with higher educational levels may be attributed to their proactiveness and willingness to seek more information and involve themselves in health decision-making (Smith et al., 2009).

Lower income level and socioeconomic status have been consistently associated with lower health literacy levels in both hearing and Deaf people. Deaf people are, however, disproportionately affected given that they experience lower literacy levels and socioeconomic status due to early language deprivation, inaccessible information, social discrimination, and limited access to employment (Batten et al., 2014; Hendry et al., 2021; Humphries et al., 2012; Jacob et al., 2022; Komesaroff, 2004; McKee et al., 2015; O’Connell, 2022). These structural inequities directly undermine health literacy by limiting opportunities to acquire, access, and apply health information across the life course. Beyond ensuring that healthcare material is in an accessible format and at the appropriate comprehension level for Deaf individuals, it is also imperative that Deaf children have timely exposure to a fully accessible language, and that more countries adopt policies that will ensure the rights of Deaf individuals are protected in all settings including ensuring the healthcare system is accessible, pushing for inclusive hiring, and protecting Deaf people against discrimination in the workplace (Grazioli et al., 2024; Humphries et al., 2012; Jacob et al., 2022; McKee et al., 2015; National Association of the Deaf, 2025).

Notably, some studies have reported relatively high levels of health literacy among Deaf participants, particularly in samples comprising Deaf college students or more highly educated individuals (Kushalnagar et al., 2018; Paludneviciene et al., 2021). This highlights the role of education and other mediating factors in shaping health literacy outcomes, which are further explored below.

### Source of health information

Deaf people often have to rely on informal information networks such as family, friends, and Deaf organizations (Chininthorn et al., 2016; Rogers et al., 2025; Siddiqi et al., 2025). Other than being due to inaccessible communication with HCPs and the healthcare system, this difference in source can also be explained by culture. The hearing population learn from their environment and from hearing conversations around them about their family medical history (Hirsh, 2003; Jacob et al., 2022). Deaf individuals, on the other hand, are deprived of these incidental learning opportunities and are often excluded from conversations at the table, a phenomenon known as “dinner table syndrome”(Chong et al., 2019; Hauser et al., 2010; Jacob et al., 2022; Pollard Jr. & Barnett, 2009). As such they look to peers and family members, as well as Deaf organizations, as there is comfort and trust, potentially a shared language among family members, a better understanding of the lived experience of Deaf individuals, as well as a reduced risk of stigma (Chininthorn et al., 2016). This should not, however, be interpreted simply as a deficit in formal health literacy. Rather, it reflects adaptive strategies Deaf people have been forced to use to navigate the inaccessible healthcare system. More concerning is the fact that these informal pathways may increase vulnerability to misinformation, particularly when healthcare information is shared without appropriate contextualization and linguistic accuracy (Rogers et al., 2025).

Our findings also suggest an increasing trend of reliance on digital technology and this preference can be due to the fact that online material can be revisited and read at one’s own pace, and would overcome the barrier caused by inaccessible HCPs who have limited time (Chininthorn et al., 2016; Kushalnagar & Kushalnagar, 2018). However, most reading materials available online are at a much higher readability level than the recommended level for Deaf people (National Institutes of Health, 2007; Neuhauser et al., 2013; Rooney et al., 2021; Weis, 2003), indicating yet another barrier that Deaf individuals might face.

The lack of information received from HCPs can be attributed to the lack of understanding of Deaf culture and barriers to communication by HCPs, resulting in Deaf individuals frequently receiving incomplete information about their diagnosis and proposed treatment (Rogers et al., 2025). Consequently, many are hesitant to approach the healthcare system and seek help (Chininthorn et al., 2016; Hommes et al., 2018; O’Riordan et al., 2024), further compounding their poorer health status (Jacob et al., 2016; O’Riordan et al., 2024). This yet again underlines the fact that health literacy challenges among Deaf people are not primarily attributable to individual deficits but rather arise from structural and communicative barriers embedded within the healthcare system. This aligns with contemporary health literacy frameworks that conceptualize health literacy as a product of interactions between individuals capacities and system-level demands (Nutbeam, 2008; Sørensen et al., 2012). There should therefore be greater emphasis on improving communication access for Deaf individuals, including, where appropriate, basic sign language training, alongside broader training in Deaf awareness and cultural competence for HCPs (Jacob et al., 2022; Young et al., 2016).

### Impact of interventions on health literacy levels

Our findings with regard to the interventions used to increase health literacy levels highlight key points; interventions must be culturally-aligned, in sign-language, and developed with the Deaf community (Folkins et al., 2005; Piao et al., 2023; Pollard Jr. et al., 2009). Adopting a community-based participatory research approach or inclusive approach will ensure that members of the Deaf community are involved in all aspects of the research process, which will enable culturally-appropriate programs to be developed, thus making them more effective and efficient (Dollinger et al., 2018; Hendricks et al., 2025; Viswanathan M et al., 2004). Another benefit to emerge from such collaborations is a deeper understanding of the community’s unique circumstances and needs, and a more accurate framework for testing and adapting best practices to the community’s actual needs versus what researchers think they need (Dollinger et al., 2018; Hendricks et al., 2025; Viswanathan M et al., 2004).

A few studies used graphically-enhanced programs and visual reinforcement, and indeed research has shown these options to be beneficial for Deaf people, who generally learn visually (McKee et al., 2015; Nikolaraizi et al., 2013; Suriani et al., 2025). Like any other language, sign language has its own word formatting and pronunciation, but it relies more on facial expressions to convey messages (National Institute on Deafness and Other Communication Disorders, 2015). The use of visual modalities align with Vygotsky’s Zone of Proximal Development framework as it meets learners where they are linguistically and developmentally (Vygotsky, 1978). Similarly, dialogic and narrative approaches, as employed by some of the included studies, have been found to be more effective as they employ story-telling, discussions, and visuals, making them more interactive and engaging, which enables information to be more digestible (Barrett et al., 2022; Fung et al., 2005; Pollard Jr. et al., 2009; Towson et al., 2016). Indeed, sign language is a dialogic language where the exchange of information happens through a give-and-take approach (Pollard Jr. et al., 2009). Personal health narratives can help in conveying complex medical information, making it more relatable, relevant, and easier to grasp (Barrett et al., 2022). This is in stark contrast to traditional didactic methods which rely heavily on linear text or spoken instruction, which is challenging for Deaf individuals who have limited literacy levels and prefer visual-based communication (Mealings et al., 2025).

It must be pointed out, however, that there is likely no one-size-fits all option, neither would there be one method that is better than the other. Based on current recommendations, several options for representation should be made available to accommodate the different learner types in formats that are accessible and easily perceptible by all learner types given that not all Deaf people are visual learners e.g., providing descriptions, tactile equivalents, spatial models, etc. (CAST, 2024). The authors in the included studies also recommended repetition to ensure retention of information, although this needs further evaluation to determine its effectiveness and over a longer period of time (Hickey et al., 2013; Jensen et al., 2013; Kumar et al., 2022; Santhosh et al., 2025).

### Strength and limitations

To our knowledge, this is the first scoping review exploring the health literacy levels of the Deaf community specifically. The sample size across the 57 included studies was also relatively large, involving more than 6,000 Deaf participants across 14 different countries, providing a clearer overall picture regarding the health literacy of the Deaf community. A few limitations were identified in this review. Firstly, there are no standardized terms on the research topic of deafness; therefore some studies that used Deaf patients could have been missed due to the wrong application of terms such as deaf, hard of hearing, or hearing loss. Similarly, diverse deaf populations are often grouped into a single category, limiting interpretability and masking important linguistic and cultural differences. This was also noted in a systematic review of the health outcomes in the Deaf by Rogers et al., where failure to specify sign language use restricted meaningful analysis of health outcomes (Rogers et al., 2024). The literature search also only included texts that were published in English potentially omitting several suitable studies, which could give an insight into a more diverse set of results. Although not always required for scoping reviews, the quality of the included studies was not formally appraised. Therefore, the findings should be interpreted as a mapping of the scope of existing evidence rather than assessing evidence of robustness. Another limitation is the limited exploration of variations in health literacy within Deaf populations such as educational level, age, and sign language proficiency. Most studies compared Deaf participants with hearing participants, rather than examining heterogeneity within Deaf communities. Finally, different measurement tools for health literacy were used for the 57 studies, which limited the possibility of a direct comparison of the results between studies. It must also be noted that while we discussed differences in knowledge between Deaf and typically hearing individuals, this was done to highlight the barriers Deaf individuals face that have led to these differences, as opposed to perpetuating the view of Deaf people as less-abled.

### Future research and recommendation

The majority of studies assessing the health literacy levels of Deaf individuals were from the US (*n =* 31) and there was a lack of data involving the Deaf community from South-East-Asia. Hence, a global overview of the health literacy among the Deaf community could not be determined, especially those from lower income countries. Lower-income populations are known to have poorer health literacy, thus it would be imperative to conduct such studies in these countries. Indeed a report from the WHO and World Bank found that there is a gap of available health services especially in countries in South East Asia and Sub-Saharan Africa (Reconstruction & and Development/The World Bank, 2017), which compounds the challenges Deaf people face with the healthcare system.

This scoping review demonstrates that research on health literacy among Deaf populations has concentrated on a limited number of health domains, with more studies on HIV/AIDS and cancer, compared to other diseases. This appears to be shaped globally by historical public health priorities, where more funding has been allocated to detection and prevention campaigns focusing on these diseases, while others like mental health are overlooked (Dzierszinski et al., 2023). Indeed, scant attention has been paid to diseases such as dementia and mental health disorders, despite the fact that studies have shown that Deaf people have a higher risk of having dementia (Liang et al., 2021), have a higher risk of dementia risk factors such as obesity and hypertension (Flower et al., 2025), and suffered more mental health conditions compared to the hearing community (Fellinger et al., 2012; Kushalnagar et al., 2019; Morisod et al., 2022). In addition, higher rates of depression among Deaf individuals may further increase the risk of dementia (Flower et al., 2025). According to a report by the National Institute for Mental Health in England, the prevalence of mental disorders in Deaf children is 40% compared to 25% in typically hearing children (National Institute for Mental Health in England, 2005). A recent study by Young et al. (Young et al., 2023) also reported higher levels of emotional and behavioral difficulties among Deaf children and adolescents compared with their hearing peers, alongside increased exposure to psychosocial stressors. This imbalance suggests that health literacy research has been reactive to specific disease agendas rather than grounded in a holistic understanding of Deaf people’s everyday health information needs. This narrow focus then risks overlooking broader determinants of health decision-making such as communication barriers, and accessibility to the healthcare system and health information. This imbalance highlights the need for further research that adopts broader, deaf-centered approaches to health literacy beyond historically prioritized conditions, and one that is grounded in community analysis to determine actual priorities (Jones et al., 2005).

The majority of the studies measured health literacy in Deaf adult populations (*n =* 18) while only three studies investigated adolescent health literacy in participants aged from 9–19 years old (Oredugba, 2004; Santhosh et al., 2025; Smith et al., 2015). The remaining studies included mixed-aged samples or did not clearly specify the age range of participants. The limited amount of investigation involving the younger Deaf population could be due to researchers aiming to find the health literacy that is acquired once self-supporting. However, this limits the scope of investigation as childhood health literacy can help to determine when gaps in knowledge arise; and this knowledge on their health literacy levels and sources of information can help in the development of appropriate interventions to increase health literacy levels in this population. This is especially crucial given that Deaf adults and Deaf children/adolescents will require different types of interventions (Gur et al., 2020; McKee et al., 2025; Smith & Samar, 2016). Further research should prioritize age-specific and longitudinal studies of health literacy in Deaf children and adolescents to inform timely and developmentally-appropriate interventions.

## Conclusion

The health literacy levels of the Deaf community were found to be lacking and poorer when compared with the hearing population. This could potentially impact their ability to detect symptoms of conditions or prevent disease, which can in turn lead to poor health outcomes. While several factors have been identified that could contribute to poor literacy levels such as poorer socioeconomic status, poor education levels, as well as communication barriers with healthcare providers, further studies involving larger sample sizes, different diseases states, more countries especially low and middle income countries, and adopting a more inclusive approach with the Deaf community; should be undertaken to fully understand the magnitude of the challenges Deaf people face with the healthcare system. Similarly, there is an urgent need for more HCPs to be culturally competent in Deaf culture and to be trained in sign language to allow for a better bi-directional healthcare consultation experience for the Deaf community.

## Supplementary Material

Appendix_A_PRISMA_table_clean_enag017

Appendix_B_Search_strategy_enag017
