# Supplementary material for: Health literacy of the Deaf community: a scoping review
Source: J Deaf Stud Deaf Educ. 2026 May 15;31(4):577–611. doi: 10.1093/jdsade/enag017 (PMC13595092; doi:10.1093/jdsade/enag017)
Supplement: Supplementary file 2 [file appendix_b_search_strategy_enag017.docx]

**Appendix B - Keywords and Search strategy**

Database used:

- Embase (Excerpta Medica Database)
- PubMed
- Cochrane
- Cumulative Index to Nursing and Allied Health Literature (CINAHL)

Other sources:

- Google
- Google scholar
- Articles’ reference list
- Gray Literature

Inclusion Criteria:

- Articles reporting on Health Literacy of the Deaf Community;
- Articles written in English;
- Publications report on the perspective of the Deaf;
- Participants specifying from the Deaf community, and who communicates primarily via sign language;
- Where participants with other disabilities are involved, the results clearly separates the Deaf participants and participants of other disabilities.

Exclusion Criteria:

- Publications that are conference preceding, conference abstract, animal studies, in vitro studies, in vivo studies, systematic reviews, meta-analysis;
- No sign language indicated in the paper;
- Where there are participants of other disabilities involved, the results of the study did not separate Deaf participants from participants of other disabilities;
- Participants with hard of hearing, not specifying from the Deaf community;
- Articles not written in English;
- Articles with no abstract or full text available;
- Articles written not in the perspective of the Deaf.

Search Terms:

Search terms are all obtained through a thorough literature review revolving the study objectives. We used MESH terms from EMBASE and PubMed, and consulted the lead researcher and a university librarian.

A. Health Literacy

“Health liter*” OR  “health competence” OR “health education” OR “health knowledge” OR “health awareness” OR “medical awareness” OR “health curriculum” OR “health attitude” OR “attitude to health” OR “health information” OR  “health numeracy” OR “medic* literacy” OR “pharmac* literacy” OR “morbidity literacy” OR “cancer literacy” OR “diabetes literacy” OR “nutritional literacy” OR “mental health literacy” OR “chronic disease literacy” OR “reproductive health literacy” OR “infectious disease literacy” OR “child health literacy” OR “ageing health literacy” OR “digital health literacy” OR “noncommunicable disease literacy” “NCD literacy” OR “ COVID-19 literacy” OR “cardiovascular literacy”

B. Deaf Community

Deaf* OR “DHH” OR “DHoH” OR “hard-of-hearing” OR “signing community” OR “Deaf signer” OR “Deaf sign user*” OR “sign language user*” OR “sign language” OR “BSL” OR “British Sign Language” OR “ASL” OR  “American Sign Language” OR “African Sign

Strategy:

1. Search A (Health Literacy keywords)

2. Search B (Deaf Community keywords)

3. Combine A with B with “AND”

4. Limit to English

5. Limit the date of publication: database inception to 31 December 2024

6. Papers imported to Endnote21 and Covidence

7. Duplicates removed

8. Title and abstract screening

9. Full text screening

**Search Terms and Search Strategy**

Databases searched:

- PubMed
- Cumulative Index to Nursing and Allied Health Literature (CINAHL)
- Excerpta Medica Database (EMBASE)
- Cochrane Central Register of Controlled Trials
- Cochrane Database of Systematic Reviews

Other sources:

- Google
- Google scholar
- Grey literature

Inclusion criteria:
- Studies reporting on either qualitative or quantitative data on the health literacy of the Deaf community.

Exclusion criteria:

(a) studies reported on the health literacy of the deaf participants, instead of the Deaf with a capital ‘D.’

(b) studies that did not separate the health literacy data of the Deaf participants from the deaf participants

(c) studies that included participants with other disabilities such as participants who were deaf-blind

(d) studies were either systematic review, meta-analyses, animal studies, in vitro or in vivo studies, conferencing preceding or conference abstracts

(e) studies that did not have abstract or full text

(f) studies that were only available in a foreign language.

Deaf Community (Search terms in each section is combined using the Boolean operator OR)

1. Deaf*
2. DHH
3. DHoH
4. hard-of-hearing
5. signing community
6. Deaf signer
7. Deaf sign user*
8. sign language user*
9. sign language
10. BSL
11. British Sign Language
12. ASL
13. American Sign Language
14. African Sign Language

Health literacy (Search terms in each section is combined using the Boolean operator OR)

1. Health liter*
2. health competence
3. health education
4. health knowledge
5. health awareness
6. medical awareness
7. health curriculum
8. health attitude
9. attitude to health
10. health information
11. Health numeracy
12. medic* literacy
13. pharmac* literacy
14. morbidity literacy
15. Cancer Literacy
16. diabetes literacy
17. nutritional literacy
18. mental health literacy
19. chronic disease literacy
20. reproductive health literacy
21. infectious disease literacy
22. child health literacy
23. ageing health literacy
24. digital health literacy
25. Noncommunicable disease literacy
26. NCD literacy
27. COVID-19 literacy
28. cardiovascular literacy

Search Strategy

1. The search terms under the Deaf Community are searched and combined using the Boolean Operator “OR”.
2. The search terms under Health literacy are searched and connected using the Boolean Operator “OR”.
3. Results from (1) and (2) are connected using the Boolean Operator (AND)
4. Results are limited to English, human studies and the time from database inception to 31st December 2024.
